# A neuronal activation correlate in striatum and prefrontal cortex of prolonged cocaine intake

**DOI:** 10.1007/s00429-017-1412-4

**Published:** 2017-04-09

**Authors:** Ping Gao, Jan C. de Munck, Jules H. W. Limpens, Louk J. M. J. Vanderschuren, Pieter Voorn

**Affiliations:** 1 Department of Anatomy and Neurosciences, Neuroscience Campus Amsterdam, VU University Medical Center, Amsterdam, The Netherlands; 2 Department of Physics and Medical Technology, VU University Medical Center, Amsterdam, The Netherlands; 3 Department of Translational Neuroscience, Brain Center Rudolf Magnus, University Medical Center Utrecht, Utrecht, The Netherlands; 4 Division of Behavioural Neuroscience, Faculty of Veterinary Medicine, Department of Animals in Science and Society, Utrecht University, Utrecht, The Netherlands

**Keywords:** Cocaine self-administration, Striatum, Medial prefrontal cortex, Orbitofrontal cortex, Immediate early gene, *Mkp1*

## Abstract

Maladaptive changes in the involvement of striatal and frontal cortical regions in drug use are thought to underlie the progression to habitual drug use and loss of cognitive control over drug intake that occur with accumulating drug experience. The present experiments focus on changes in neuronal activity in these regions associated with short-term (10 days) and long-term (60 days) self-administration of cocaine. Quantitative in situ hybridization for the immediate early gene *Mkp1* was combined with statistical parametric mapping to assess the distribution of neuronal activity. We hypothesized that neuronal activity in striatum would increase in its dorsal part and that activity in frontal cortex would decrease with prolonged cocaine self-administration experience. Expression of *Mkp1* was profoundly increased after cocaine self-administration, and the magnitude of this effect was greater after short-term compared to long-term self-administration. Increased neuronal activity was seen in both dorsal and ventral sectors of the striatum after 10 days exposure to cocaine. However, enhanced activity was restricted to dorsomedial and dorsocentral striatum after 60 days cocaine self-administration. In virtually all medial prefrontal and most orbitofrontal areas, increased expression of *Mkp1* was observed after 10 days of cocaine taking, whereas after 60 days, enhanced expression was restricted to caudal parts of medial prefrontal and caudomedial parts of orbitofrontal cortex. Our data reveal functional changes in cellular activity in striatum and frontal cortex with increasing cocaine self-administration experience. These changes might reflect the neural processes that underlie the descent from recreational drug taking to compulsive cocaine use.

**Electronic supplementary material:**

The online version of this article (doi:10.1007/s00429-017-1412-4) contains supplementary material, which is available to authorized users.

## Introduction

Drug addiction is a chronic relapsing brain disorder hallmarked by persistent drug seeking and taking that occurs even in the face of adverse consequences (American Psychiatric Association 2000, 2013; Leshner 1997; Volkow and Li 2004). The apparent loss of control over drug-directed behavior is thought to find its cause in a maladaptive transition from goal-directed to habitual drug-directed behavior in combination with breakdown of prefrontal cortical cognitive control over drug intake as a result of prolonged and excessive drug use (Jentsch and Taylor 1999; Everitt and Robbins 2005; Hyman et al. 2006; Koob and Volkow 2010; Pierce and Vanderschuren 2010; Goldstein and Volkow 2011).

The neural substrate of the process of habit formation that is thought to be pathologically affected by prolonged drug use lies within the striatum. Whereas the ventral striatum (VS) is associated with reinforcement signaling and the medial portion of dorsal striatum (DS) with goal-directed behavior, the lateral part of DS has been implicated in habit learning (Carelli 2002; Corbit and Janak 2007; Yin et al. 2008; Roesch et al. 2009; Stalnaker et al. 2010; Gremel and Costa 2013; Burton et al. 2015). On basis of these findings, Everitt and Robbins (2005) have proposed a shift in functional involvement in drug use from ventromedial to dorsolateral striatum as drug experience progresses. For cocaine, support for this hypothesis comes from behavioral studies in laboratory animals (Everitt and Robbins 2016) and from studies examining changes in neural activation and dopaminergic neurotransmission in non-human primates and human addicts (Garavan et al. 2000; Letchworth et al. 2001; Porrino et al. 2004; Volkow et al. 2006; Wong et al. 2006). The cognitive ability to limit drug intake involves a complex process of impulse control, planning and decision-making in which medial prefrontal cortex (mPFC) and orbitofrontal cortex (OFC) play a central role (London et al. 2000; O’Neill et al. 2001; Franklin et al. 2002; Wilson et al. 2004; Volkow et al. 2005; Goldstein et al. 2009; Perry et al. 2011; Wilcox et al. 2011; Mihindou et al. 2013; Lucantonio et al. 2014). Malfunction of these cortical regions may lead to loss of control over drug seeking and taking, a suggestion that is supported by substantial evidence concerning the transition to drug addiction (Jentsch and Taylor 1999; Porrino and Lyons 2000; Volkow and Fowler 2000; Goldstein and Volkow 2002, 2011; Steketee 2003; Everitt et al. 2007; Kalivas 2008; Feil et al. 2010; Lucantonio et al. 2012; Chen et al. 2013; Kasanetz et al. 2013; Limpens et al. 2015).

There is evidence to suggest actual changes in neuronal activity associated with accumulating cocaine self-administration experience. These changes were predominantly seen in VS and restricted parts of DS after limited access to cocaine, and they spread to more dorsal parts of DS after long-term self-administration (Letchworth et al. 2001; Porrino et al. 2004). In OFC and mPFC, differences between the effects of acute or limited cocaine exposure and extended cocaine self-administration have also been reported (Porrino and Lyons 2000). Studies using expression of immediate early genes (IEGs) as markers of neuronal activity after acute, limited and extended (self-) administration of cocaine have reported rather complex patterns of region- and exposure time-dependent changes in expression of several IEGs (Daunais et al. 1993, 1995; Larson et al. 2010; Zahm et al. 2010; Besson et al. 2013; Fumagalli et al. 2013; Gao et al. 2017). Some of these patterns appear in accordance with hypothesized changes in functional involvement of striatal sectors or cortical regions, as described above, whereas others do not. At this point, however, it is difficult to interpret these data since our knowledge on the effects of prolonged cocaine self-administration on IEG expression is very limited indeed (Larson et al. 2010; Besson et al. 2013). Therefore, in the present study the corollaries of short (10 days)- and long-term (60 days) self-administration of cocaine for IEG expression were assessed in rat striatum and prefrontal cortex. These time points were chosen because they are considered to be equivalent to early and advanced stages of the addiction process. Thus, it is well established that cocaine self-administration patterns stabilize after approximately ten sessions (e.g., De Vries et al. 1998; Deroche-Gamonet et al. 2002; Veeneman et al. 2012; Gao et al. 2017), whereas signs of addiction-like behavior typically emerge after about 50 self-administration sessions (Deroche-Gamonet et al. 2004; Vanderschuren and Everitt 2004; Kasanetz et al. 2010; Limpens et al. 2014; for review see; Vanderschuren et al. 2017). The IEG mitogen-activated protein kinase phosphatase 1 (*Mkp1* or *Dusp1*) was selected as neuronal activity marker.

An enzyme that dephosphorylates extracellular signal regulated kinase (*ERK*), Jun N-terminal kinase (*JNK*) and p38 mitogen-activated protein kinase, *Mkp1* serves as a negative feedback regulator in MAPK pathway activity (Wancket et al. 2012; Korhonen and Moilanen 2014). Numerous extracellular stimuli, including exposure to drugs of abuse, have been found to induce *Mkp1* transcription, either directly or indirectly via activation of the MAPK pathway (Takaki et al. 2001; Ujike et al. 2002; Miller and Marshall 2005; Wancket et al. 2012; Korhonen and Moilanen 2014; Gao et al. 2017). The MAPK pathway has been shown to play an important role in cocaine and opiate addiction processes, probably by modulating long-term synaptic plasticity (Lu et al. 2006; Pascoli et al. 2011; García-Pardo et al. 2016). Recently, using quantitative PCR, we reported increased expression of *Mkp1* after short- and long-term self-administration of cocaine (Gao et al. 2017). These experiments, however, did not allow for high resolution mapping of activated neurons that may unveil subregional changes (Gao et al. 2017). Therefore, quantitative in situ hybridization was used in the present experiments to assess *Mkp1* expression levels after 10 or 60 days of self-administration of cocaine or sucrose. Furthermore, in addition to conventional quantitative analysis of anatomically defined striatal and cortical subregions of interest we also designed a mapping technique based on statistical parametric mapping (SPM) (Friston 1995) avoiding predetermined delineations, since activational patterns do not necessarily respect conventional anatomical boundaries. We expected to find a stronger functional involvement of VS compared to DS after short-term cocaine self-administration, and activational patterns more restricted to DS after long-term cocaine exposure. For mPFC and OFC, we expected to see increased IEG expression after short-term cocaine self-administration, followed by decreases after long-term cocaine experience.

## Methods

### Subjects

Male Wistar rats (*n* = 48) (Charles River, Sulzfeld, Germany) weighing 320–380 g were housed individually in Macrolon cages (*L* = 40 cm, *W* = 25 cm, *H* = 18 cm) under controlled conditions (temperature = 20–21 °C, 55 ± 15% relative humidity) and a reversed 12 h light–dark cycle (lights on at 19:00 h.). Each subject received 20 g of laboratory chow (SDS Ltd, UK) per day and free access to water, which was sufficient to maintain body weight and growth. Self-administration sessions were carried out between 09:00 and 18:00 h. for 5 days a week. All experiments were approved by the Animal Ethics Committee of Utrecht University and were conducted in agreement with Dutch laws (Wet op de Dierproeven, 1996) and European regulations (Guideline 86/609/EEC).

### Apparatus

Subjects were trained and tested in operant conditioning chambers (*L* = 29.5 cm, *W* = 32.5 cm, *H* = 23.5 cm; Med Associates, Georgia, VT, USA). The chambers were placed in light- and sound-attenuating cubicles equipped with a ventilation fan. Each chamber was equipped with two 4.8-cm wide retractable levers, placed 11.7 cm apart and 6.0 cm from the grid floor. A cue light (28 V, 100 mA) was present above each active lever and a white house light (28 V, 100 mA) was located on the opposite wall. Sucrose pellets (45 mg, formula F, Research Diets, New Brunswick, NJ, USA) were delivered at the wall opposite to the levers via a dispenser. An infusion pump placed on top of the cubicles controlled cocaine infusions. During the cocaine self-administration sessions, polyethylene tubing ran from the syringe placed in the infusion pump via a swivel to the cannula on the animals’ back. In the operant chamber, tubing was shielded with a metal spring. Experimental events and data recording were controlled by procedures written in MedState Notation using MED-PC for Windows.

### Surgery

Rats allocated to the cocaine self-administration group were anaesthetized with ketamine hydrochloride (0.075 mg/kg, i.m.) and medetomidine (0.40 mg/kg, s.c.) and supplemented with ketamine as needed. A single catheter was implanted in the right jugular vein aimed at the left vena cava. Catheters (Camcaths, Cambridge, UK) consisted of a 22 g cannula attached to silastic tubing (0.012 ID) and fixed to nylon mesh. The mesh end of the catheter was sutured subcutaneously (s.c.) on the dorsum. Carprofen (50 mg/kg, s.c.) was administered once before and twice after surgery. Gentamycin (50 mg/kg, s.c.) was administered before surgery and for 5 days post-surgery. Animals were allowed 10 days to recover from surgery.

### Cocaine and sucrose self-administration procedures

Rats (*n* = 8 in both 10- and 60-days experiments) were trained to self-administer cocaine under a fixed ratio-1 (FR-1) schedule of reinforcement. During the self-administration sessions, two levers were present, an active lever and an inactive lever. The left or right position of the active and inactive levers was counterbalanced for individual animals. Pressing the active lever resulted in the infusion of 0.25 mg of cocaine in 0.1 mL of saline over 5.6 s, retraction of the levers and switching off of the house light. During the infusion, a cue light above the lever was switched on, followed by a 20 s time-out period after which the levers were reintroduced and the house light illuminated. The time-out period was changed to 3 min after five training sessions to increase the session length. The session ended after 2 h or if animals had obtained 40 cocaine infusions, whichever occurred first. Responding on the inactive lever had no programmed consequences. After each self-administration session, intravenous catheters were flushed with a gentamycin-heparin-saline solution to maintain the patency of the catheters.

The training procedure for the rats in the sucrose group (*n* = 8 in both 10- and 60-days experiments) was similar to that for cocaine self-administration, with the exception that each response on the active lever resulted in delivery of a sucrose pellet. Subjects in the control group (*n* = 8 in both 10- and 60-days experiments) were also exposed to the self-administration box. Each response on the active lever resulted in illumination of the cue light for 5.6 s.

### Tissue dissection

After the last training session, rats were moved back to their home cage, and decapitated after 30 min. Brains were quickly removed and immediately frozen in cold isopentane, and stored at −80 °C. Fourteen micrometer thick coronal sections were cut at −25 °C in a cryostat (Leica CM 1950), then mounted on SuperFrost^®^ Plus glass slides (Menzel*-*Gläser, Braunschweig, Germany) and stored at −80 °C.

### In situ hybridization

The synthesis of *Mkp1* cRNA probe included total RNA extraction, cDNA synthesis using TaqMan® reverse transcription reagents kit (Applied Biosystems, Branchburg, NJ, USA), DNA amplification using Phusion*®* High*-*Fidelity PCR Kit (Finnzymes, New England Biolabs, Inc.). *Mkp1* primers contained either a T7 or T3 RNA polymerase promoter sequence and their sequences are: sense 5′-AATTAACCCTCACTAAAGGGTGAAGCAGAGGCGGAGTATT-3′ and antisense 5′-GTAATACGACTCACTATAGGGCAAGGGAAGAAACTGGCTCA-3′. PCR amplification conditions were 98 °C/2 min, followed by 30 cycles of 98 °C/20 s, 66 °C/40 s, 72 °C/1 min, and a final 72 °C/10 min. Finally, cRNA probes were generated using MAXIscript^®^ T3⁄T7 In Vitro Transcription Kit (Ambion Inc., Austin, TX). *Mkp1-*probes were labeled by incorporating digoxigenin-labeled UTP (Roche Diagnostics GmbH, Mannheim, Germany).

For in situ hybridization (ISH), rat brain sections were fixed in 4% paraformaldehyde, acetylated using acetic anhydride (0.25% acetic anhydride in 1.5% triethanolamine buffer), delipidated and dehydrated. *Mkp1* digoxigenin-labeled RNA probes (5 ng/section) were diluted in 120 µl hybridization buffer (50% formamide, 4× SSC, tRNA 2.5 g/l, 2% 50× Denhardt’s reagent, 10% Dextran) and applied to slides holding four sections. Hybridization was performed at 60 °C in humid chambers for 18 h. Post-hybridization stringency washings were 1× SSC at 60 °C (2 × 30 min), followed by a RNase A treatment in 2× SSC buffer at 37 °C for 15 min. After another washing in 1× SSC at 60 °C (30 min) sections were incubated for 1 h in 1× SSC at room temperature. Subsequently, sections were exposed to blocking solution (TRIS-NaCl buffer with 1% blocking powder, Roche Diagnostics GmbH, Mannheim, Germany) for 1 h at room temperature and incubated with anti-digoxigenin-alkaline phosphatase antibody (1:2500, Roche Diagnostics GmbH, Mannheim, Germany) at 4 °C overnight. Alkaline phosphatase was visualized using NBT/BCIP (Roche Diagnostics GmbH, Mannheim, Germany) as a substrate. Color development was in the dark at room temperature overnight and the enzymatic reaction was stopped in TRIS-NaCl buffer with EDTA (1.212% TRIS, 0.876% NaCl, 0.0372% EDTA, pH = 7.5).

### Data analysis of in situ hybridization

Quantification of hybridized *Mkp1-*probe was carried out using an MCID Elite image system (Interfocus Imaging Ltd., Linton, UK). Coronal sections were digitized using an objective magnification of 5X on a Leica DM/RBE photomicroscope connected to a digital camera (Evolution™ MP Color camera, Media Cybernetics, Rockville, Maryland). A composite image of striatum and cortex was made utilizing the MCID tiling tool and a motorized stage. The segmentation of *Mkp1*-positive cells from background was performed using an algorithm combining several point operators and spatial filters, aiming at detecting local changes in gray level, and thus, produce a measuring template for objects. Images went through histogram equalization, smoothing (low-pass filter, kernel size 7 × 7), and subtraction steps, and the positive cells were finally detected according to their size and shape (van Kerkhof et al. 2014). To improve visualization, contrast was enhanced of the in situ hybridization images in Figs. 4a–d and 5a–f.

For each region, the analysis was performed at two anatomical levels from anterior and posterior. For mPFC and OFC, two levels are Bregma +3.7 and +2.7 mm, and for striatum, Bregma +1.7 and + 1.0 mm. The outlines of brain (sub)regions were defined according to Van De Werd and Uylings (2008). Parameters that were measured included the number of positive cells in each subregion, the subregional surface area, the integrated optical density (OD) of each individual cell body (representing labeling intensity) and the individual *X*–*Y* coordinates of all cells. Changes in number of positive cells were expressed as changes in density (number of cells/mm^2^). Labeling intensity was expressed as OD value averaged over all cells per subregion per animal. Subsequently, cell density and OD values were used for statistical parametric mapping (SPM) analysis (see below).

### Statistical analysis

Data was analyzed using SPSS software 20 (IBM, New York, NY, USA). A repeated measures ANOVA was used for analyzing cocaine/sucrose self-administration, with group as a between-subject factor and self-administration duration as a within-subject factor. To determine whether the effects of short-term cocaine self-administration on the *Mkp1* mRNA differ from that after long-term administration in cortex and striatum, for each subregion the density and the intensity of *Mkp1-*positive cells in the cocaine and the sucrose groups were normalized to control and further analyzed using a two-way ANOVA with group and self-administration duration as two factors. A Bonferroni post hoc test or Student’s *t* test was used where appropriate. A corrected *p* value <0.05 was considered to indicate statistical significance.

Anatomical (sub)regions (including anterior and more posterior subregions) were selected on basis of known differences in neuroanatomical connections and/or functional involvement in drug addiction. Statistical comparisons involved planned comparisons restricted to specific regions, the outcome of which was not generalized to the entire brain. Furthermore, data was analyzed using two independent methods, i.e., the above method and the statistical parametric mapping method described below.

### Statistical parametric mapping (SPM) analysis

To investigate the cocaine and sucrose-induced differences in the neuronal activation patterns in cortex and striatum, the numbers of positive cells, cellular labeling intensity, and *X*–*Y* coordinates were used to produce statistical parametric maps.

**Fig. 1:**
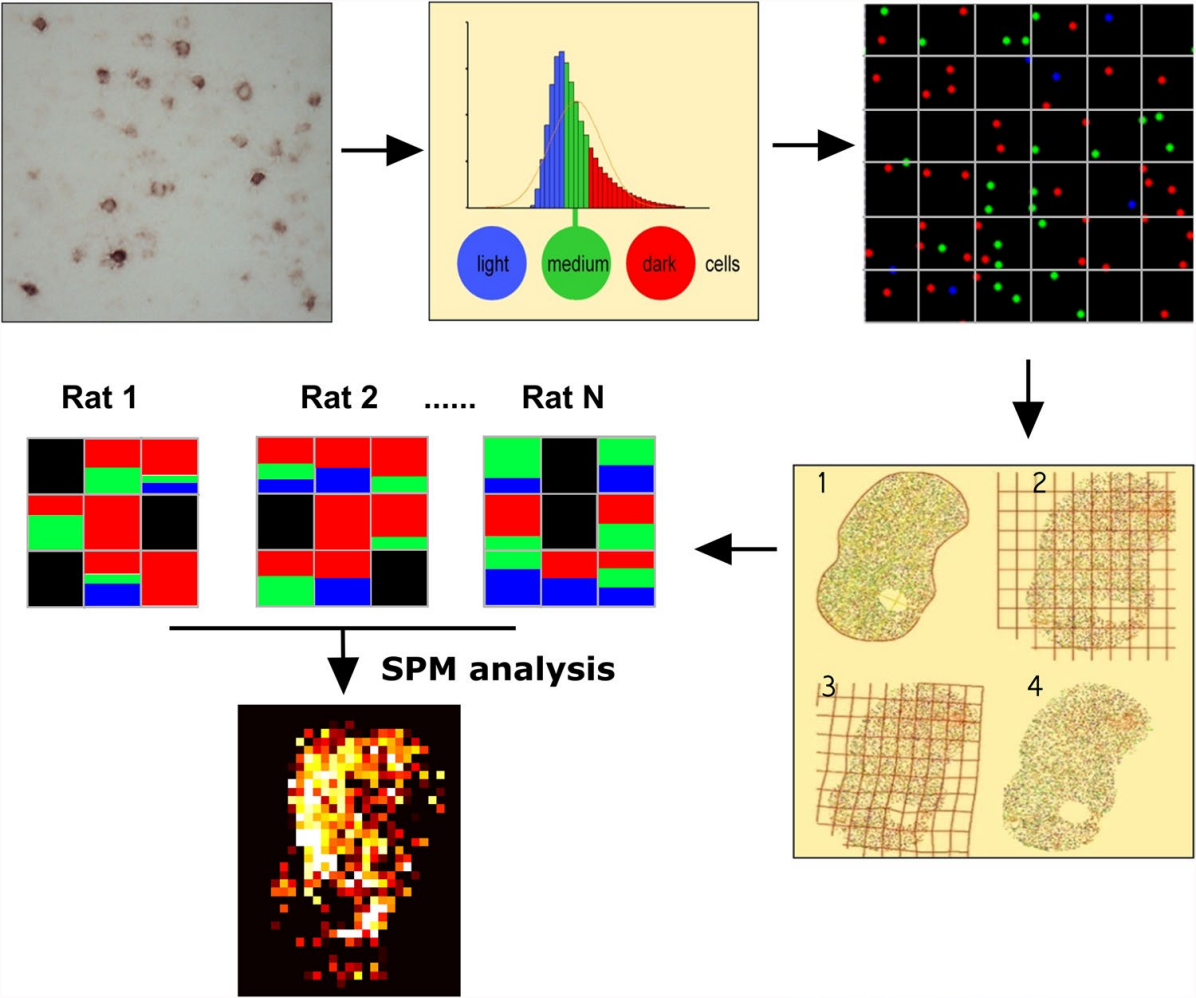
Main steps in procedure for SPM analysis. Cellular labeling intensity and *x*–*y* position of *Mkp1*-cells were recorded (*top left*) and cells were binned into three color-coded groups based on labeling intensity (*top middle*). Next, the color-coded cells were plotted (*top right*) and the anatomical region of interest with plotted cells (*bottom right*, 2) was warped to a reference image (*bottom right*, 1). The warped image is shown in 4 (*bottom right*); the transformation is illustrated by deformation of the raster overlying the reference image in 2 and the warped image in 3. Finally (*bottom, left*), density images were generated from the warped images by counting the number of cells for each labeling intensity per square in a raster image. These images were used for performing statistic parametric mapping

In this method, the response of *Mkp1-*cells to the experimental conditions was compared in a manner that takes into account cellular labeling intensity and cell density. First, the entire population of cells at the anterior or posterior anatomical levels of striatum or cortex in the sucrose and cocaine self-administration groups of animals was divided into three groups on basis of the 33th and 66th percentile values of cellular labeling intensity (OD) in the control group (Fig. 1). The cells in the three bins were assigned a numerical and color code (i.e., red for dark, blue for light and green for medium intensity) and plotted using the previously determined *X*–*Y* coordinates in a RGB color image utilizing MatLab (Fig. 1). Each cell was represented by a pixel kernel with approximately the size of striatal neurons, viz. 10 × 10 µm (11 × 11 pixels). Subsequently, a reference, average outline of the striatum or cortical regions, was superimposed on the plotted cell image for use as a template in a warping procedure that served to transform sections from all individual animals to the same outline (Fig. 1). A set of approximately 25 fiducial points (for striatum) was used in the warping procedure, ranging from anatomical landmarks, such as the ventral tip of the lateral ventricle, to calculated landmarks like the geometrical center of gravity of particular subregions. Care was taken to exclude deformation of cellular pixel kernels during warping. Next, the warped sections were used for SPM analysis (Fig. 1).

The warping process yielded for each section of each animal an image that was subsequently converted into an array listing *X*–*Y* coordinates of the individual, labeling intensity-coded activated cells in a common coordinate frame. These arrays were converted into density images, by defining a rectangular raster and counting the number of cells of each type per square. The optimal raster size is a trade-off between resolution and sensitivity. We chose a raster size of 150 µm × 150 µm. To increase sensitivity and to account for reminiscent errors due to imperfect image warping, the density images were spatially blurred with a Gaussian filter with a standard deviation of 200 µm. The statistical comparison of two groups of animals over multiple pixels, and in different cell sizes, is equivalent to standard fMRI data analysis methods (Worsley and Friston 1995), where statistical comparisons are made between images made in resting and active conditions. Such statistical comparisons are based on a linear regression, wherein the available data is modeled as a sum regressors of interest and regressors acting as confounds. A partial correlation coefficient is derived for each pixel, of which the statistical significance is computed, and corrections are made for multiple testing of null hypothesis over different pixels. To apply the standard fMRI method for detecting the contrast between (e.g.,) cocaine and sucrose self-administration animals, we derived the following regressors. First, the images of the * N*_C_ “cocaine” animals (number of images in the cocaine group) were stacked onto N_S_ images of “sucrose” animals (number of images in the sucrose group). Each image contains three values (number of light cells, number of medium cells and the number of dark cells). In this way, we obtain for each pixel a 3(*N*_C_ + N_*S*_) dimensional vector *d*_*j*_ of measurements. The following regressors are defined as confounds:

$$C_{j}^{1} = \left\{ {\begin{array}{ll} 1 & \quad {{\text{if }}j{\text{ refers to a light pixel}}} \\ {0,} & \quad {{\text{otherwise}}} \\ \end{array} } \right.$$

$$C_{j}^{2} = \left\{ {\begin{array}{ll} 1 & \quad {{\text{if }}j{\text{ refers to a medium pixel}}} \\ {0,} & \quad {{\text{otherwise}}} \\ \end{array} } \right.$$

$$C_{j}^{3} = \left\{ {\begin{array}{ll} 1 & \quad {{\text{if }}j{\text{ refers to a dark pixel}}} \\ {0,} & \quad {{\text{otherwise}}} \\ \end{array} } \right.$$

These regressors refer to the hypothesis that the cell density is constant (independent of the condition of the animal). Three regressors of interest, referring to a possible difference in condition, are defined as

$$S_{j}^{1} = \left\{ {\begin{array}{ll} 1 & {{\text{if}}~j~{\text{refers}}~{\text{to}}~{\text{a}}~{\text{light}}~{\text{pixel}}~{\text{in}}~{\text{``cocaine''}}~{\text{animal}}} \\ {0,} & {{\text{otherwise}}~} \\ \end{array} } \right.$$

$$S_{j}^{2} = \left\{ {\begin{array}{ll} 1 & {{\text{if}}~j~{\text{refers}}~{\text{to}}~{\text{a}}~{\text{medium}}~{\text{pixel}}~{\text{in}}~{\text{``cocaine''}}~{\text{animal}}} \\ {0,} & {{\text{otherwise}}~} \\ \end{array} } \right.$$

$$S_{j}^{3} = \left\{ {\begin{array}{ll} 1 & {{\text{if}}~j~{\text{refers}}~{\text{to}}~{\text{a}}~{\text{dark}}~{\text{pixel}}~{\text{in}}~{\text{``cocaine''}}~{\text{animal}}} \\ {0,} & {{\text{otherwise}}~} \\ \end{array} } \right.$$

The data vector is modeled as

$${d_j}=\sum\limits_{k=1,2,3} {{\alpha _k}} S{}_j^k+\sum\limits_{k=1,2,3} {{\beta _k}} C{}_j^k+{\eta _j},$$

where *η*_*j*_ is assumed to be Gaussian white noise. The ML principle is applied to estimate the regression coefficients *α*_*k*_ and *β*_*k*_ from the data and a partial correlation coefficient *r*, that expresses how well the data can be reconstructed using the regressors of interest, when the confounds are removed from the data. This partial correlation coefficient has a one-to-one relation to the *F* statistic, and therefore its significance can be tested in a straightforward way.

This statistical analysis is applied for each pixel independently. Although background pixels were skipped, there are many pixels involved (*N* ~ 400 for striatum), and therefore there is a large probability that some significant effects will be found at some pixels, just by chance alone. To correct for these kinds of type I errors, the observed probabilities were converted into a False Detection Rate (Benjamini and Hochberg 1995), which gives the expected fraction of pixels with a type I error. The activation images shown here (Fig. 8) are thresholded at a maximum FDR of 20%. The meaning of this value indicates that 20% of the pixels declared active, is in reality a false positive.

We present the results of the statistical mapping method by creating a pseudo-anatomical gray scale image consisting of the average of all non-smoothed density images. Superimposed on this gray scale image, we plot all pixels with an FDR of 0.1% or larger in a color scale corresponding to the partial correlation coefficient *r*.

## Results

### Cocaine and sucrose self-administration

**Fig. 2:**
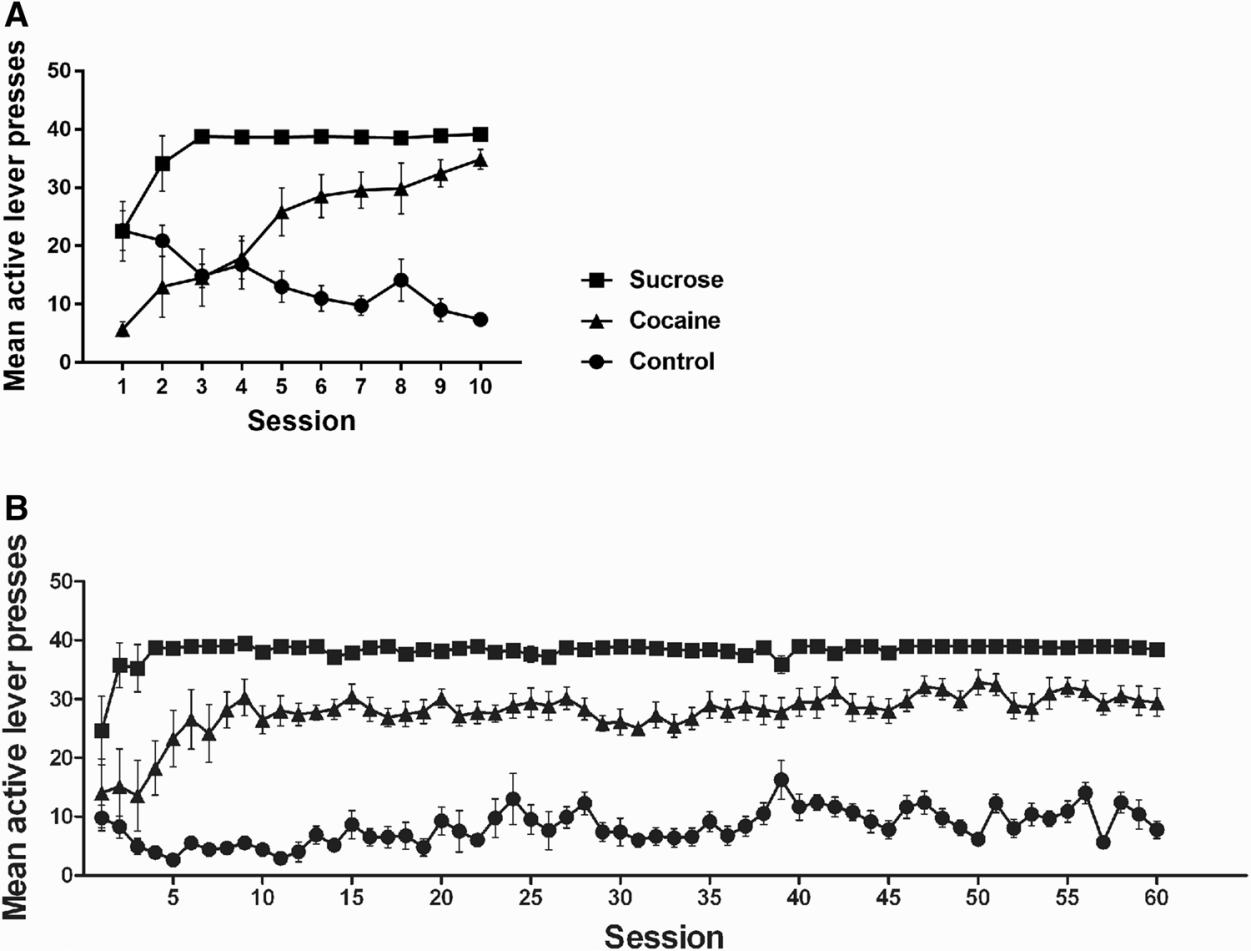
Short-term (10 days, **a**) and long-term (60 days, **b**) self-administration of cocaine or sucrose. Data is presented as mean ± SEM number of active lever presses over animals per group

Rats were trained to self-administer cocaine or sucrose for either 10 or 60 days under a FR1 schedule of reinforcement (Fig. 2). The number of active lever presses represents the total amount of rewards acquired per session. Repeated measures ANOVA revealed that there was a main effect of group on the active lever responses in the 10-days [*F*(2, 20) = 45.85, *p* < 0.001] and the 60-days experiments [*F*(2, 20) = 179.41, *p* < 0.001]. Post-hoc tests showed significant differences between the cocaine and control [10 days: *p* = 0,003, 60 days: *p* < 0.001], the cocaine and sucrose [10 days: *p* < 0.001, 60 days: *p* < 0.001], as well as the sucrose and control groups [10 days: *p* < 0.001, 60 days: *p* < 0.001] for both experiments. A main effect of self-administration duration on the active lever presses was observed in 10 days [*F*(4.05, 80.99) = 5.22, *p* = 0.001] and 60 days experiments [*F*(6.16, 123.15) = 6.02, *p* < 0.001]. The interactions between group and self-administration duration were significant [10 days: *F*(8.10, 80.99) = 9.96, *p* < 0.001; 60 days: *F*(12.32, 123.15) = 2.59, *p* = 0.004]. Comparing the numbers of active lever presses between the first and the final sessions, there were significant increases in the cocaine (10 days: *t* = −13.55, *df* = 14, *p* < 0.001; 60 days: *t* = −2.44, *df* = 7.89, *p* = 0.041) and sucrose groups (10 days: *t* = −3.24, *df* = 7.008, *p* = 0.014; 60 days: *t* = −2.358, *df* = 7.073, *p* = 0.05). For the control groups we observed a decrease after 10 days (*t* = 4.32, *df* = 7.98, *p* = 0.003) and no changes after 60 days (*t* = 0.769, *df* = 14, *p* = 0.455). The average number of inactive lever presses in the cocaine and sucrose groups was lower than four per session from the fifth until the final self-administration session for both experiments (data not shown). The number of active lever presses was significantly higher than the number of responses to the inactive lever in the cocaine group [10 day: *F*(1, 12) = 58.82, *p* < 0.001; 60 days: *F*(1, 12) = 212.31, *p* < 0.001] and sucrose group [10 day: *F*(1, 14) = 1006.92, *p* < 0.001; 60 days: *F*(1, 14) = 10097.27, *p* < 0.001], but not in the control group [10 day: *F*(1, 14) = 0.01, *p* = 0.918; 60 days: *F*(1, 14) = 2.39, *p* = 0.144].

### Cell density

**Fig. 3:**
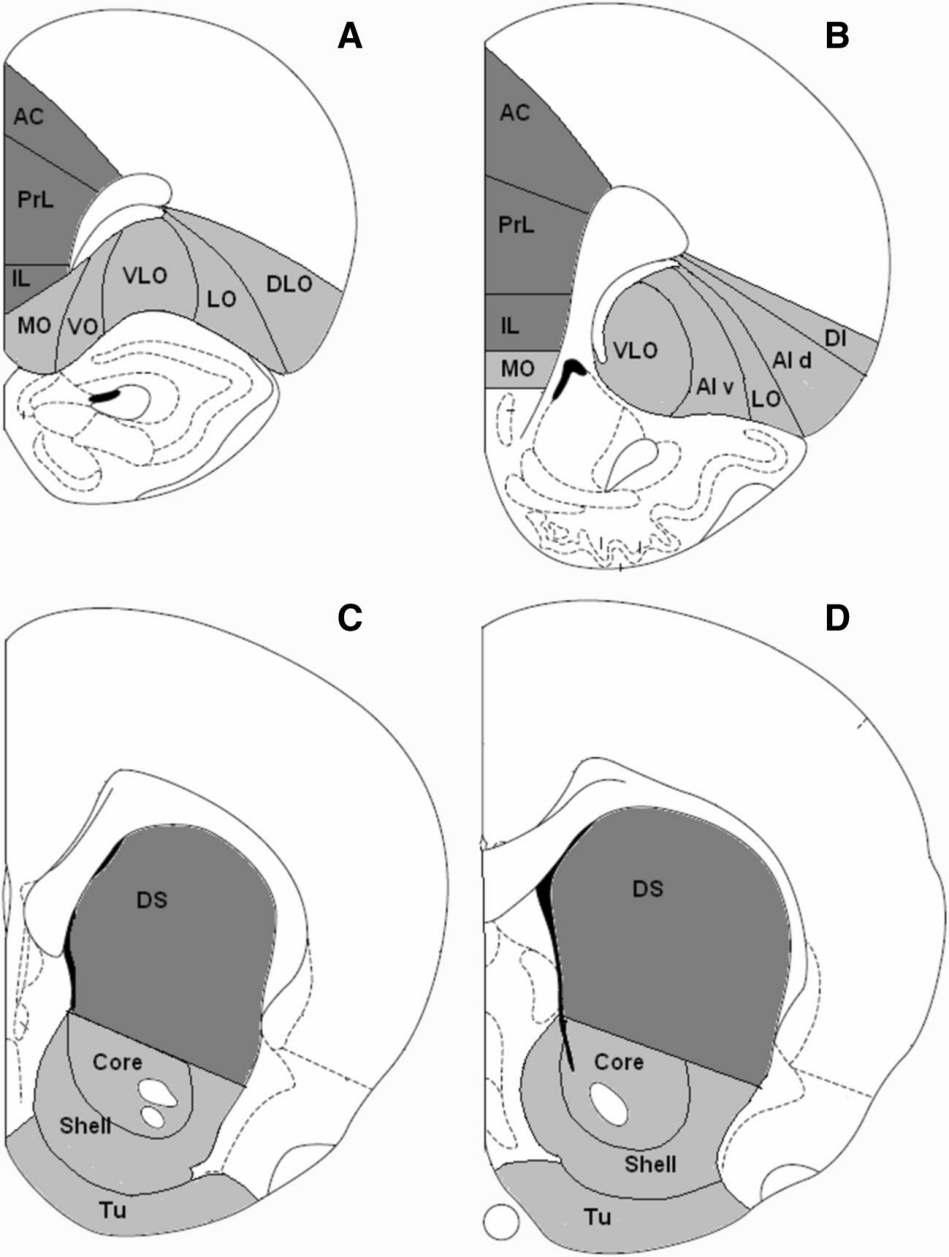
Regions of interest in coronal sections of rat brain at anterior and posterior levels. For mPFC and OFC, two levels are Bregma +3.7 mm (**a**) and +2.7 mm (**b**). For striatum, two levels are Bregma +1.7 mm (**c**) and + 1.0 mm (**d**). *AC* anterior cingulate cortex, *PrL* prelimbic cortex, *IL* infralimbic cortex, *MO* medial OFC, *VO* ventral OFC, *VLO* ventral lateral OFC, *LO* lateral OFC, *DLO* dorsal lateral OFC, *AIv* agranular insular cortex–ventral part, *AId* agranular insular cortex–dorsal part, *DI* dysgranular insular cortex, *DS* dorsal striatum, *Tu* olfactory tubercle

*Mkp1*-positive neurons were measured in 11 regions of interest in cortex and four regions of interest in striatum at two anterior-posterior levels (for most regions) (Fig. 3). Compared to the control and sucrose groups, cocaine self-administration produced major changes in the density of the *Mkp1-*labeled neurons (Tables 1, S1).

**Table 1:** Results of two-way ANOVA in subregions of striatum, mPFC and OFC with group and self-administration duration (i.e., 10 vs. 60 days of self-administration) as independent variables

Region	Subregion	Group (*F* ratio; *p* value)		Self-administration duration (*F* ratio; *p* value)		Interaction (*F* ratio; *p* value)	
		Density (CD)	Intensity (OD)	Density (CD)	Intensity (OD)	Density (CD)	Intensity (OD)
Striatum (Anterior)	DS	*F*(2.40) = 54.39	*F*(2.40) = 22.04	*F*(1.40) = 0.22	*F*(1.40) = 1.46	*F*(2.40) = 2.11	*F*(2.40) = 0.42
		***p*** < 0.001*	***p*** < 0.001*	*p* = 0.640	*p* = 0.233	*p* = 0.134	*p* = 0.656
	Core	*F*(2.40) = 39.59	*F*(2.40) = 17.53	*F*(1.40) = 17.98	*F*(1.40) = 3.75	*F*(2.40) = 22.78	*F*(2.40) = 0.96
		***p*** < 0.001*	***p*** < 0.001*	***p*** < 0.001*	*p* = 0.060	***p*** < 0.001*	*p* = 0.392
	Shell	*F*(2.40) = 16.16	*F*(2.40) = 11.56	*F*(1.40) = 3.52	*F*(1.40) = 0.39	*F*(2.40) = 6.08	*F*(2.40) = 0.11
		***p*** < 0.001*	***p*** < 0.001*	*p* = 0.068	*p* = 0.531	***p*** = 0.005*	*p* = 0.894
	Tu	*F*(2.40) = 10.45	*F*(2.40) = 5.94	*F*(1.40) = 13.78	*F*(1.40) = 0.06	*F*(2.40) = 9.72	*F*(2.40) = 0.37
		***p*** < 0.001*	***p*** = 0.006*	***p*** = 0.001*	*p* = 0.797	***p*** < 0.001*	*p* = 0.687
Striatum (Posterior)	DS	*F*(2.40) = 39.00	*F*(2.40) = 54.23	*F*(1.40) = 23.76	*F*(1.40) = 1.64	*F*(2.40) = 16.31	*F*(2.40) = 1.76
		***p*** < 0.001*	***p*** < 0.001*	***p*** < 0.001*	*p* = 0.207	***p*** < 0.001*	*p* = 0.185
	Core	*F*(2.40) = 20.41	*F*(2.40) = 43.29	*F*(1.40) = 16.85	*F*(1.40) = 1.71	*F*(2.40) = 11.53	*F*(2.40) = 0.43
		***p*** < 0.001*	***p*** < 0.001*	***p*** < 0.001*	*p* = 0.198	***p*** < 0.001*	*p* = 0.647
	Shell	*F*(2.40) = 14.79	*F*(2.40) = 45.24	*F*(1.40) = 4.33	*F*(1.40) = 16.00	*F*(2.40) = 4.76	*F*(2.40) = 4.69
		***p*** < 0.001*	***p*** < 0.001*	***p*** = 0.044*	***p*** < 0.001*	***p*** = 0.014*	***p*** = 0.015*
	Tu	*F*(2.40) = 6.59	*F*(2.40) = 9.79	*F*(1.40) = 0.91	*F*(1.40) = 0.01	*F*(2.40) = 1.12	*F*(2.40) = 0.55
		***p*** = 0.003*	***p*** < 0.001*	*p* = 0.345	*p* = 0.945	*p* = 0.336	*p* = 0.576
mPFC (Anterior)	AC	*F*(2.40) = 6.20	*F*(2.40) = 34.38	*F*(1.40) = 9.92	*F*(1.40) = 5.40	*F*(2.40) = 14.52	*F*(2.40) = 6.97
		***p*** = 0.004*	***p*** < 0.001*	***p*** = 0.003*	***p*** = 0.025*	***p*** < 0.001*	***p*** = 0.003*
	PrL	*F*(2.40) = 6.82	*F*(2.40) = 35.23	*F*(1.40) = 9.51	*F*(1.40) = 9.57	*F*(2.40) = 9.31	*F*(2.40) = 8.79
		***p*** = 0.003*	***p*** < 0.001*	***p*** = 0.004*	***p*** = 0.004*	***p*** < 0.001*	***p*** = 0.001*
	IL	*F*(2.40) = 9.93	*F*(2.40) = 27.30	*F*(1.40) = 7.27	*F*(1.40) = 0.59	*F*(2.40) = 7.69	*F*(2.40) = 2.72
		***p*** < 0.001*	***p*** < 0.001*	***p*** = 0.010*	*p* = 0.444	***p*** = 0.001*	*p* = 0.077
mPFC (Posterior)	AC	*F*(2.39) = 10.43	*F*(2.39) = 20.57	*F*(1.39) = 9.91	*F*(1.39) = 11.45	*F*(2.39) = 3.68	*F*(2.39) = 3.26
		***p*** < 0.001*	***p*** < 0.001*	***p*** = 0.003*	***p*** = 0.002*	***p*** = 0.034*	***p*** = 0.049*
	PrL	*F*(2.39) = 18.63	*F*(2.39) = 22.11	*F*(1.39) = 15.40	*F*(1.39) = 12.99	*F*(2.39) = 5.60	*F*(2.39) = 3.93
		***p*** < 0.001*	***p*** < 0.001*	***p*** < 0.001*	***p*** = 0.001*	***p*** = 0.007*	***p*** = 0.028*
	IL	*F*(2.39) = 9.83	*F*(2.39) = 14.17	*F*(1.39) = 0.76	*F*(1.39) = 4.76	*F*(2.39) = 0.31	*F*(2.39) = 1.43
		***p*** < 0.001*	***p*** < 0.001*	*p* = 0.388	***p*** = 0.035*	*p* = 0.730	*p* = 0.252
OFC (Anterior)	MO	*F*(2.40) = 13.99	*F*(2.40) = 36.67	*F*(1.40) = 13.07	*F*(1.40) = 7.77	*F*(2.40) = 13.62	*F*(2.40) = 7.04
		***p*** < 0.001*	***p*** < 0.001*	***p*** = 0.001*	***p*** = 0.008*	***p*** < 0.001*	***p*** = 0.002*
	VO	*F*(2.40) = 7.14	*F*(2.40) = 25.72	*F*(1.40) = 8.51	*F*(1.40) = 10.44	*F*(2.40) = 19.15	*F*(2.40) = 5.15
		***p*** = 0.002*	***p*** < 0.001*	***p*** = 0.006*	***p*** = 0.002*	***p*** < 0.001*	***p*** = 0.010*
	VLO	*F*(2.40) = 5.24	*F*(2.40) = 36.00	*F*(1.40) = 1.11	*F*(1.40) = 8.34	*F*(2.40) = 8.19	*F*(2.40) = 6.08
		***p*** = 0.009*	***p*** < 0.001*	*p* = 0.296	***p*** = 0.006*	***p*** = 0.001*	***p*** = 0.005*
	LO	*F*(2.40) = 6.50	*F*(2.40) = 32.47	*F*(1.40) = 0.64	*F*(1.40) = 9.37	*F*(2.40) = 7.90	*F*(2.40) = 5.24
		***p*** = 0.004*	***p*** < 0.001*	*p* = 0.426	***p*** = 0.004*	***p*** = 0.001*	***p*** = 0.010*
	DLO	*F*(2.40) = 1.83	*F*(2.40) = 31.06	*F*(1.40) = 2.84	*F*(1.40) = 12.22	*F*(2.40) = 4.67	*F*(2.40) = 6.52
		*p* = 0.172	***p*** < 0.001*	*p* = 0.099	***p*** = 0.001*	***p*** = 0.015*	***p*** = 0.004*
OFC (Posterior)	MO	*F*(2.39) = 13.50	*F*(2.39) = 17.08	*F*(1.39) = 2.04	*F*(1.39) = 0.79	*F*(2.39) = 3.51	*F*(2.39) = 0.70
		***p*** < 0.001*	***p*** < 0.001*	*p* = 0.161	*p* = 0.379	***p*** = 0.039*	*p* = 0.500
	VLO	*F*(2.39) = 16.63	*F*(2.39) = 38.23	*F*(1.39) = 1.78	*F*(1.39) = 4.40	*F*(2.39) = 4.58	*F*(2.39) = 1.21
		***p*** < 0.001*	***p*** < 0.001*	*p* = 0.190	***p*** = 0.042*	***p*** = 0.016*	*p* = 0.309
	AIv	*F*(2.39) = 12.28	*F*(2.39) = 40.31	*F*(1.39) = 5.52	*F*(1.39) = 14.40	*F*(2.39) = 4.50	*F*(2.39) = 5.04
		***p*** < 0.001*	***p*** < 0.001*	***p*** = 0.024*	***p*** = 0.001*	***p*** = 0.017*	***p*** = 0.011*
	LO	*F*(2.39) = 9.51	*F*(2.39) = 23.86	*F*(1.39) = 6.20	*F*(1.39) = 2.92	*F*(2.39) = 3.61	*F*(2.39) = 1.32
		***p*** < 0.001*	***p*** < 0.001*	***p*** = 0.017*	*p* = 0.095	***p*** = 0.036*	*p* = 0.279
	AId	*F*(2.39) = 1.48	*F*(2.39) = 30.28	*F*(1.39) = 0.12	*F*(1.39) = 4.86	*F*(2.39) = 0.06	*F*(2.39) = 3.34
		*p* = 0.240	***p*** < 0.001*	*p* = 0.722	***p*** = 0.033*	*p* = 0.941	***p*** = 0.046*
	DI	*F*(2.39) = 0.50	*F*(2.39) = 12.38	*F*(1.39) = 3.96	*F*(1.39) = 14.48	*F*(2.39) = 1.08	*F*(2.39) = 4.21
		*p* = 0.606	***p*** < 0.001*	*p* = 0.054	***p*** < 0.001*	*p* = 0.347	***p*** = 0.022*

Cell density (i.e., number of cells per surface area) and labeling intensity were dependent variables. A corrected *p* value <0.05 implies statistical significance and is indicated with an asterisk

### Striatum

**Fig. 4:**
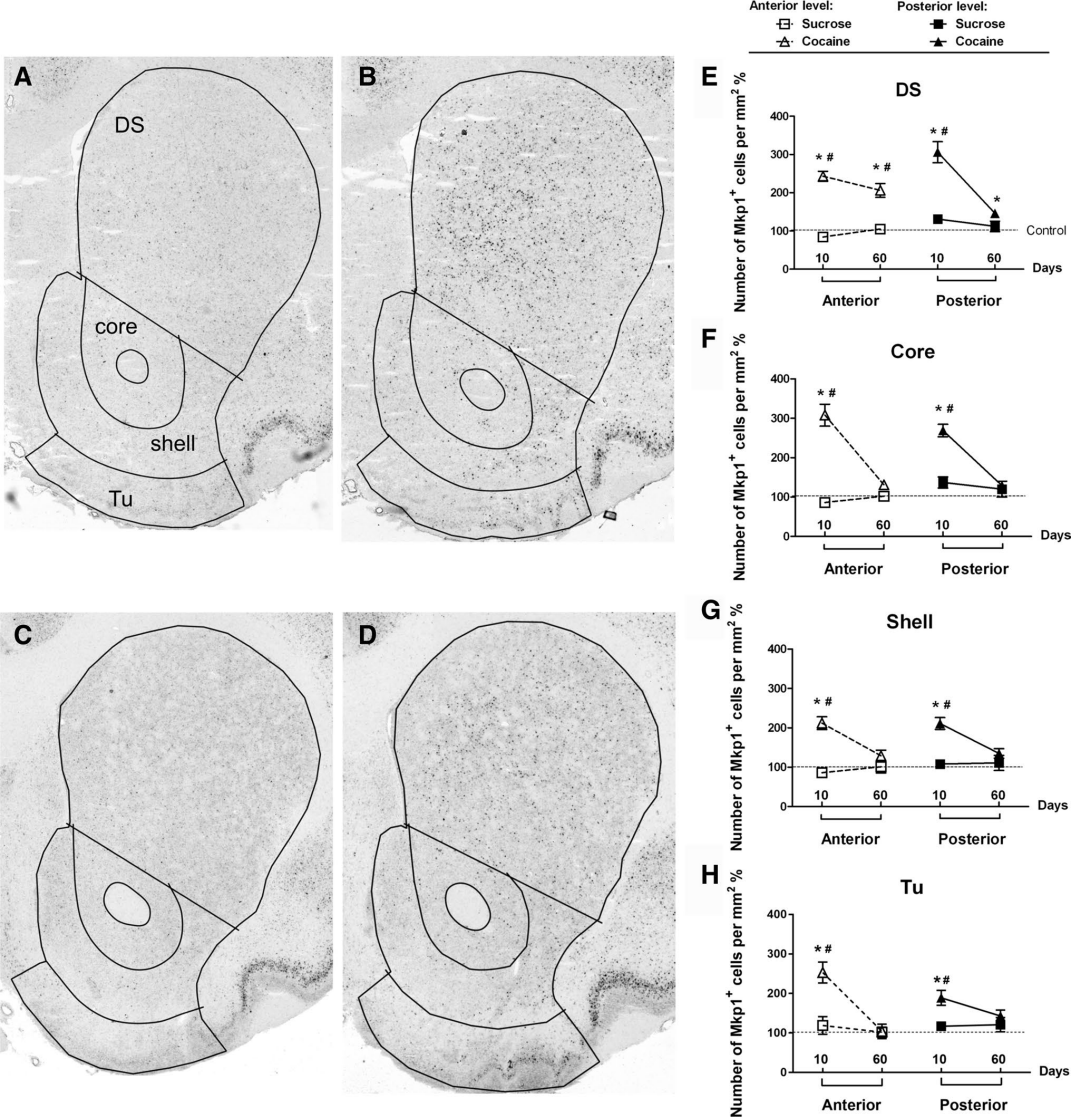
Micrographs of representative hybridized coronal sections through striatum (at posterior level) showing the distribution of *Mkp1*-positive cells after 10 days (**a** control, **b** cocaine) and 60 days (**c** control, **d** cocaine) self-administration. Superimposed lines delineate regions of interest for quantification. **e**–**h** Densities of *Mkp1-*positive cells in individual striatal subregions at the two anterior-posterior anatomical levels after 10 and 60 days of self-administration. Values are presented as mean ± SEM percentage of cells/mm^2^ in the control groups. *p < 0.05, significant differences between cocaine and control groups; ^#^ *p* < 0.05, significant differences between cocaine and sucrose groups. *Tu* olfactory tubercle

In striatum, 10 days of cocaine self-administration induced a marked increase in the density of *Mkp1-*positive cells in both the DS and VS (Fig. 4a, b). Likewise, after 60 days exposure to cocaine, an increase was observed, albeit of lower magnitude (Fig. 4c, d). In the anterior level of striatum, a main effect of group was seen in each subregion. A main effect of self-administration duration (10 vs. 60 days) was seen in core and olfactory tubercle (Tu), but not in DS and shell (Table 1). A significant interaction between group and self-administration duration was seen in core, shell, and Tu, but not in DS (Table 1). Post-hoc tests showed that compared to the control (Con) and sucrose (Suc) groups, the density of *Mkp1*-positive cells in the cocaine group (Coc) increased significantly in DS in both the 10 (Coc vs. Con: *p* < 0.001, Coc vs. Suc: *p* < 0.001) and 60 days experiments (Coc vs. Con: *p* < 0.001, Coc vs. Suc: *p* < 0.001) (Fig. 4e, left panel). In core, shell, and Tu, increases were observed after 10 days (Coc vs. Con: core, *p* < 0.001, shell, *p* < 0.001, Tu, *p* < 0.001; Coc vs. Suc: core, *p* < 0.001, shell, *p* < 0.001, Tu, *p* = 0.002) but not after 60 days (Fig. 4f–h, left panels).

Similar results were obtained in the posterior level of striatum. A main effect of group was seen in all striatal subregions (Table 1). Except in Tu, a main effect of self-administration duration was seen in other subregions (Table 1). The interaction between group and self-administration duration was also significant in DS, core, and shell, but not in Tu (Table 1). Post-hoc tests showed that, in DS, 10 days of cocaine self-administration significantly enhanced the density of *Mkp1*-positive cells when compared to control (*p* < 0.001) and sucrose self-administration (*p* < 0.001). After 60 days, a significant difference was only observed between the cocaine and control groups (*p* = 0.024) (Fig. 4e, right panel). In VS, 10 days of cocaine self-administration significantly increased the density of *Mkp1*-positive cells, when compared to the control (core: *p* < 0.001, shell: *p* < 0.001, Tu: *p* = 0.003) and the sucrose groups (core: *p* < 0.001, shell: *p* < 0.001, Tu: *p* = 0.009) (Fig. 4f–h, right panels). In contrast, after 60 days no differences were seen in these three subregions (Fig. 4f–h, right panels).

In summary, 10 days of cocaine self-administration increased the density of *Mkp1*-positive cells in both DS and VS, whereas after 60 days such effects were only observed in DS. Compared to the 10 days cocaine self-administration, the magnitude of increases in the density of *Mkp1*-positive cells was much lower in the cocaine group in the 60 days experiment. In addition, no effects of sucrose self-administration were observed on the density of *Mkp1*-positive cells.

### Medial prefrontal cortex

**Fig. 5:**
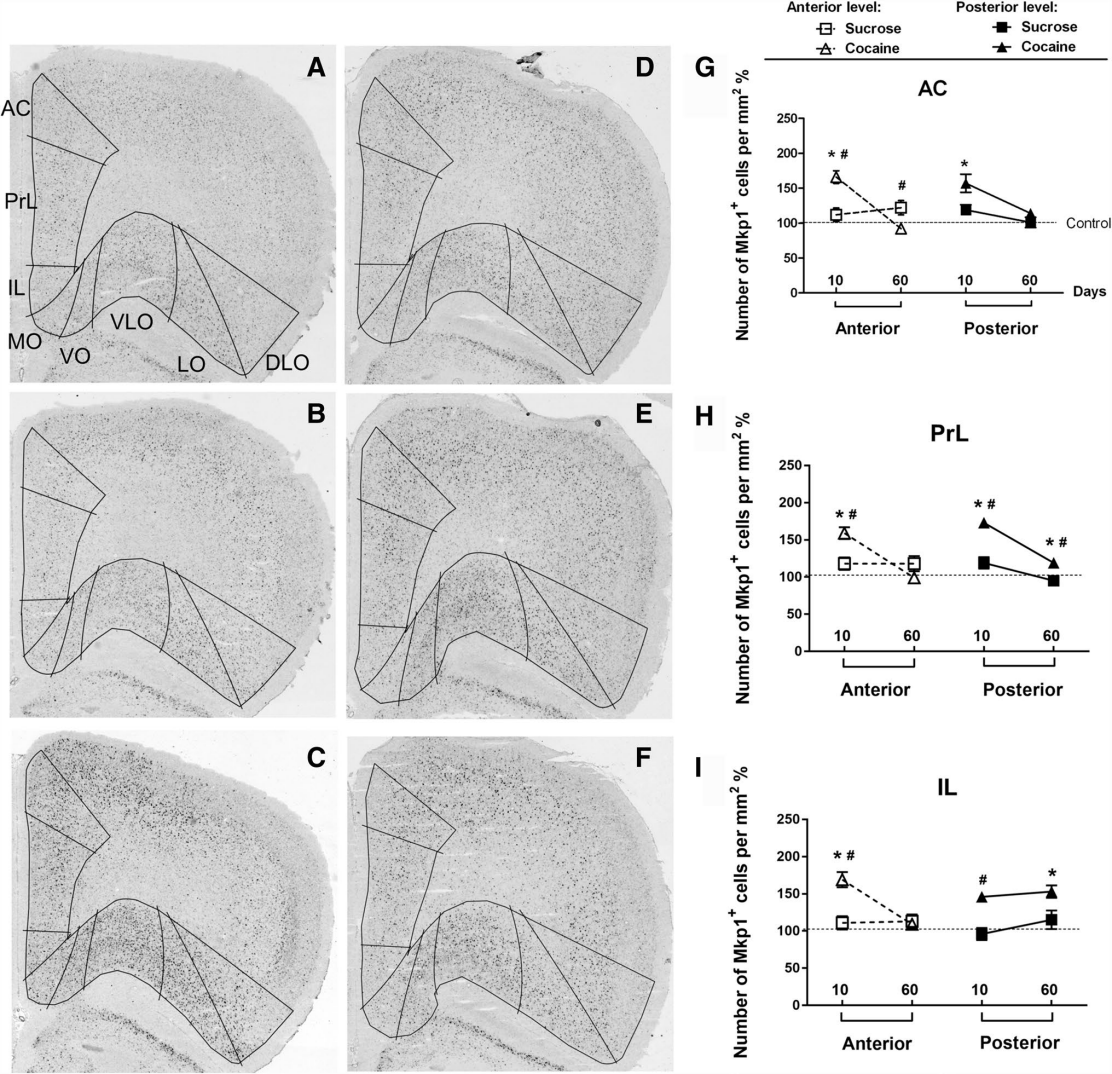
Micrographs of representative hybridized coronal sections of mPFC and OFC (at anterior level) showing the distribution of *Mkp1*-positive cells after 10 days (**a** control, **b** sucrose, **c** cocaine) and 60 days (**d** control, **e** sucrose, **f** cocaine) self-administration. Superimposed lines delineate regions of interest for quantification. Differences between micrographs from the sucrose groups at 10 (**b**) and 60 (**e**) days of self-administration appear larger than they actually are after normalization to control, as presented in **g**–**i**. Normalization was used since signal levels were higher in 60 days controls (**d**) compared to 10 days controls (**a**). **g**–**i** Densities of *Mkp1-*positive cells in individual subregions of mPFC at the two anterior-posterior anatomical levels after 10 and 60 days of self-administration. Values are presented as mean ± SEM percentage of cells/mm^2^ in the control groups. *p < 0.05, significant difference between cocaine and control groups; ^#^ *p* < 0.05, significant difference between cocaine and sucrose groups. For abbreviations, see Fig. 3

The density of *Mkp1*-positive cells in medial prefrontal cortex (mPFC) was examined in two anterior to posterior levels of anterior cingulate (AC), prelimbic (PrL) and infralimbic (IL) cortices (Fig. 3a, b). Figure 5a–f shows representative images of the *Mkp1* in situ hybridization in sections taken at the anterior level of mPFC. In anterior mPFC, two-way ANOVA revealed a main effect of group, of self-administration duration and significant interactions between the two factors in AC, PrL and IL (Table 1). Post-hoc tests showed that, in the 10-days experiment, cocaine significantly increased the density of *Mkp1*-positive cells in all subregions when compared to control (AC, *p* < 0.001, PrL, *p* < 0.001, IL, *p* < 0.001) and sucrose groups (AC, *p* = 0.002, PrL, *p* = 0.006, IL, *p* = 0.002) (Fig. 5g–i, left panels). In contrast, after 60 days, only in AC there was a significant difference in the density of *Mkp*-positive cells between the cocaine and sucrose groups (*p* = 0.032) (Fig. 5g, left panel). No changes were observed in PrL or IL (Fig. 5h, i, left panels).

In posterior mPFC (Fig. 3b), there was a main effect of group in AC, PrL, and IL, and a main effect of self-administration duration in AC and PrL, but not in IL (Table 1). The interaction between group and self-administration duration was significant for AC and PrL but not for IL (Table 1). Post-hoc tests showed that in the 10-days experiment, cocaine self-administration enhanced the density of *Mkp1*-labeled cells in AC and PrL, when compared to the control (AC: *p* = 0.002, PrL: *p* < 0.001, IL: *p* = 0.065). Enhancements were seen in PrL and IL when compared to sucrose self-administration (AC: *p* = 0.050, PrL: *p* = 0.005, IL: *p* = 0.049, Fig. 5g, h, right panels). In the 60-day experiment, cocaine-induced increases in the density of *Mkp1*-labeled cells were found in PrL and IL, when compared to control (AC: *p* = 0.245, PrL: *p* = 0.049, IL: *p* = 0.006), while an increase was seen only in PrL when compared to the sucrose group (AC: *p* = 0.296, PrL: *p* = 0.011, IL: *p* = 0.056, Fig. 5g–i, right panels). Finally, no effect of sucrose self-administration was observed at this level (Fig. 5g–i, right panels).

In summary, 10 days of cocaine self-administration increased the density of *Mkp1*-positive cells in both anterior and posterior mPFC, while 60-day cocaine self-administration showed limited effects on the density of *Mkp1-positive* cells in the posterior mPFC. In the anterior level of mPFC, the magnitude of changes in the density of *Mkp1*-positive cells after 60 days was much lower, when compared to the 10 days of cocaine self-administration.

### Orbitofrontal cortex

**Fig. 6:**
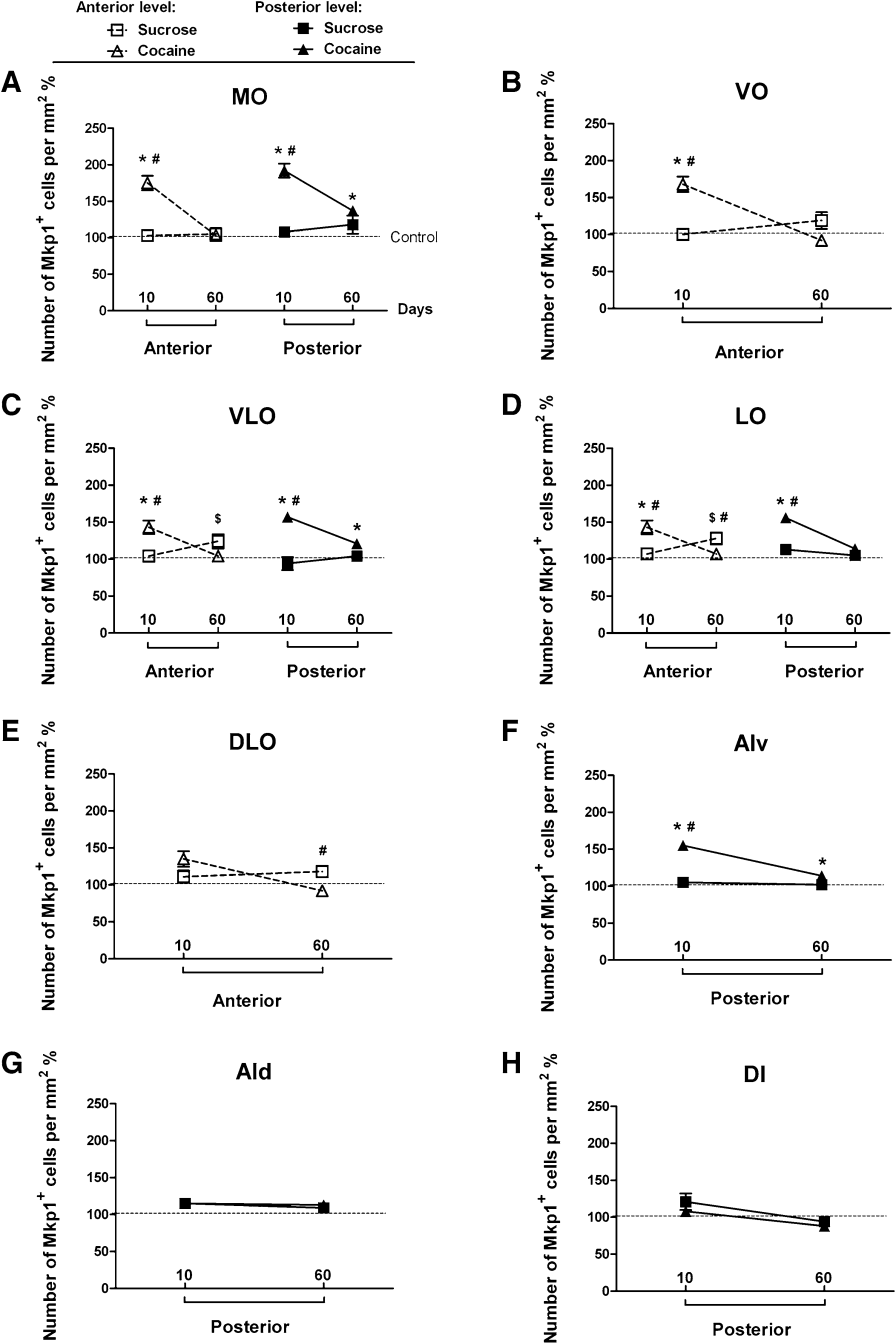
*Mkp1* cell densities in subregions of OFC after short- and long-term cocaine and sucrose self-administration. Quantification was performed at two anterior-posterior anatomical levels for MO, VLO and LO. The other regions were present only in a single anatomical level. Values are presented as mean ± SEM percentage of cells/mm^2^ in the control groups. *p < 0.05, significant difference between cocaine and control groups; ^#^ *p* < 0.05, significant difference between cocaine and sucrose groups. ^$^ *p* < 0.05, significant difference between sucrose and control groups. For abbreviations, see Fig. 3

Cocaine and sucrose self-administration affected the *Mkp1*-positive cells in orbitofrontal cortex (OFC, for anatomical structures see Fig. 3a, b) in a complex way. Figure 5a–f show representative images of the *Mkp1* in situ hybridization at the anterior level of OFC. In this region, a two-way ANOVA revealed a main effect of group in medial orbitofrontal (MO), ventral orbitofrontal (VO), ventral lateral orbitofrontal (VLO) and lateral orbitofrontal (LO) cortices but not in the dorsolateral orbitofrontal cortex (DLO) (Table 1). There was a main effect of self-administration duration in MO and VO, but not in other subregions (Table 1). The interactions between group and self-administration duration were significant in all subregions (Table 1). Post-hoc tests showed that in the 10-day experiment, the density of *Mkp1*-labeled cells was increased significantly in the cocaine group in MO, VO, VLO and LO, but not in DLO, when compared to the control (MO: *p* < 0.001, VO: *p* < 0.001, VLO: *p* = 0.003, LO: *p* = 0.006, DLO: *p* = 0.103) and sucrose groups (MO: *p* < 0.001, VO: *p* < 0.001, VLO: *p* = 0.007, LO: *p* = 0.021, DLO: *p* = 0.421) (Fig. 6a, c, d, left panels; b, e). In contrast, after 60 days, differences were seen between cocaine and sucrose groups in LO (*p* = 0.040) and DLO (*p* = 0.014) (Fig. 6d, e). Interestingly, significant up-regulation of the density of *Mkp1*-labeled cells was also observed in the sucrose self-administration animals in VLO (*p* = 0.044) and LO (*p* = 0.004) when compared to the control (Fig. 6c, d, left panel).

For ease of discussion, the ventral part of agranular insular cortex (AIv), the dorsal part of agranular insular cortex (AId), and the dysgranular insular cortex (DI) will be described as part of the posterior OFC under investigation (Fig. 3b). Two-way ANOVA showed a main effect of group on the density of *Mkp1*-positive cells in MO, VLO, AIv and LO, but not in AId and DI (Table 1). A main effect of self-administration duration was seen only in AIv and LO (Table 1). The interactions between group and self-administration duration were significant in MO, VLO, AIv, and LO (Table 1). Post-hoc testing showed that, after 10 days exposure, cocaine significantly enhanced the density of *Mkp1*-labeled cells in MO, VLO, LO and AIv, when compared to the control (MO: *p* = 0.001, VLO: *p* = 0.002, LO: *p* = 0.006, AIv: *p* = 0.003) and sucrose groups (MO: *p* = 0.004, VLO: *p* = 0.001, LO: *p* = 0.048, AIv: *p* = 0.009) (Fig. 6a, c, d, right panels; f). After 60 days of self-administration, cocaine increased the density of *Mkp1*-labeled cells in the medial portion of posterior OFC, i.e., MO (*p* = 0.046), VLO (*p* = 0.017) and AIv (*p* = 0.043) when compared to control (Fig. 6a, c, right panels; f). In addition, no effects of sucrose self-administration were seen in the posterior OFC.

To summarize, 10 days of cocaine self-administration increased the density of *Mkp1*-labeled cells in both anterior and posterior OFC. After 60 days, this effect was much smaller. Interestingly, in anterior OFC, 60 days of sucrose self-administration enhanced the density of *Mkp1*-labeled cells. After 60 days of self-administration, cocaine-induced *Mkp1* signals were observed mainly in the medial parts of OFC (at the posterior level), while sucrose-induced-*Mkp1* signals were located in the lateral parts of OFC (at the anterior level). In both 10 -and 60-day experiments, neither cocaine nor sucrose self-administration had effects in the most lateral part of posterior OFC.

### Intensity of cellular response

**Fig. 7:**
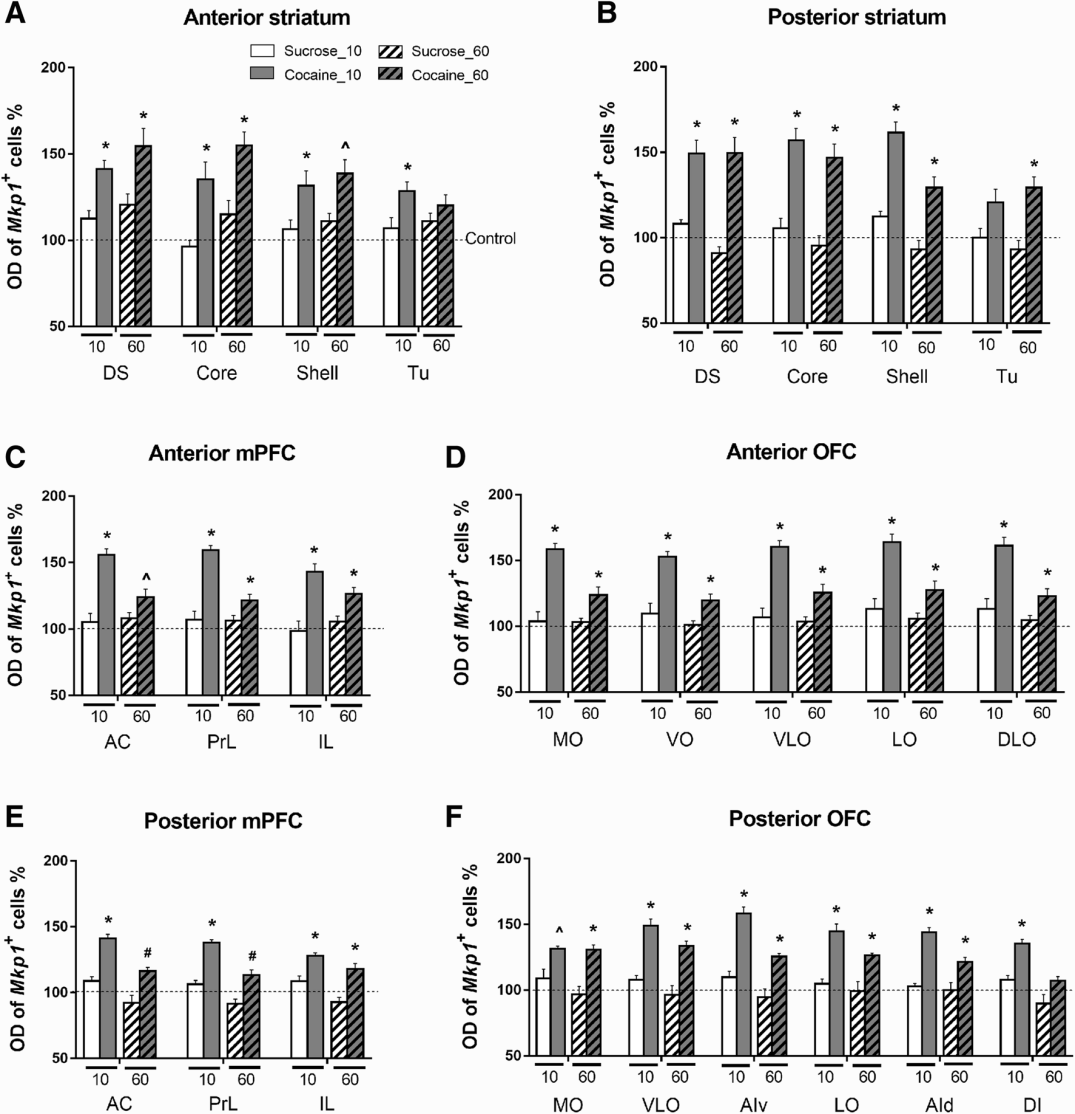
Individual labeling intensity of *Mkp1* cells in all regions of interest after short- and long-term cocaine and sucrose self-administration. Values are presented as mean ± SEM percentage of OD of *Mkp1-*positive cells (*Mkp1* ^+^ *)* in the control groups. *p < 0.05, significant difference between cocaine group and the other two groups (control group and sucrose group); ^^^ *p* < 0.05, significant difference between cocaine and control groups; ^#^ *p* < 0.05, significant difference between cocaine and sucrose groups. For abbreviations, see Fig. 3

Cocaine and sucrose self-administration affected not only the density of *Mkp1-*positive cells but also the labeling intensity of the positive cells. In striatum, two-way ANOVA showed that there was a main effect of group on the staining intensity of *Mkp1*-positive cells in all subregions (Table 1). A main effect of self-administration duration was observed only in the shell at the posterior striatum level (Table 1). The interaction between group and self-administration duration was also significant in the posterior shell (Table 1). Post-hoc testing revealed that, in the anterior striatum, after 10 days exposure the intensity of *Mkp1-positive* cells in the cocaine group was significantly higher than in the control (DS, *p* < 0.001; core, *p* = 0.007; shell, *p* = 0.007; Tu, *p* = 0.006) and sucrose groups (DS, *p* = 0.001; core, *p* = 0.003, shell, *p* = 0.034; Tu, *p* = 0.042) (Fig. 7a). After 60 days, significant differences were seen in DS (*p* = 0.001), core (*p* = 0.001) and shell (*p* = 0.011) but not in Tu (*p* = 0.216) between the cocaine and control groups. Significant differences between the cocaine and sucrose groups were seen in DS (*p* = 0.041) and core (*p* = 0.015), but not in shell (*p* = 0.076) and Tu (*p* = 0.709) (Fig. 7a). In the posterior striatum, significant differences between cocaine and the other two groups were found in DS (*p* < 0.001 for both), core (*p* < 0.001 for both), and shell (*p* < 0.001 for both), but not in Tu (Coc vs. Con, *p* = 0.198; Coc vs. Suc, *p* = 0.197) in the 10-day experiment (Fig. 7b). In the 60-day experiment, significant differences were seen in all subregions between the cocaine and control groups (DS, *p* < 0.001; core, *p* < 0.001; shell, *p* = 0.003; Tu, *p* = 0.002), and between the cocaine and sucrose groups (DS, *p* < 0.001; core, *p* < 0.001; shell, *p* < 0.001; Tu, *p* < 0.001) (Fig. 7b).

In mPFC, two-way ANOVA showed that there was a main effect of group on the intensity of *Mkp1*-positive cells in all subregions (Table 1). A main effect of self-administration duration was observed in the AC and PrL in anterior mPFC and the AC, PrL, and IL in posterior mPFC (Table 1). The interaction between group and self-administration duration was significant in the AC and PrL in both the anterior and posterior mPFC (Table 1). Post-hoc tests showed that, in the anterior mPFC, the intensity of *Mkp1*-positive cells was significantly higher after cocaine self-administration in all subregions when compared to control (AC, *p* < 0.001; PrL, *p* < 0.001; IL, *p* < 0.001) and to sucrose self-administration (AC, *p* < 0.001; PrL, *p* < 0.001; IL, *p* < 0.001) in the 10-day experiment (Fig. 7c). In the 60-day experiment, cocaine self-administration significantly increased the intensity of *Mkp1*-positive cells in all subregions when compared to control (AC, *p* = 0.003; PrL, *p* = 0.003; IL, *p* = 0.001) when compared to sucrose self-administration, significant increases in the cocaine group were observed in PrL and IL (AC, *p* = 0.06; PrL, *p* = 0.041; IL, *p* = 0.01) (Fig. 7c). In posterior mPFC, 10-day cocaine exposure significantly increased the intensity of *Mkp1*-positive cells in all subregions when compared to both the control (AC, *p* < 0.001; PrL, *p* < 0.001; IL, *p* = 0.003) and the sucrose groups (AC, *p* < 0.001; PrL, *p* < 0.001; IL, *p* = 0.003) (Fig. 7e). In contrast, 60-day cocaine exposure significantly increased the intensity of *Mkp1*-positive cells in IL, but not in AC and PrL, when compared to control (AC, *p* = 0.195; PrL, *p* = 0.169; IL, *p* = 0.038). Significant differences were seen in all three subregions between the cocaine and the sucrose groups (AC, *p* = 0.027; PrL, *p* = 0.01; IL, *p* = 0.003) (Fig. 7e). Furthermore, no difference was observed between the sucrose and control groups in mPFC.

In OFC, a main effect of group was observed on the intensity of *Mkp1*-positive cells in all subregions (Table 1). A main effect of self-administration duration was seen in all subregions in anterior OFC and in VLO, AIv, AId, and DI in posterior OFC (Table 1). A significant interaction between group and self-administration duration was seen in all subregions of anterior OFC and in AIv, AId, and DI in posterior OFC (Table 1). Post-hoc tests showed that, in anterior OFC, the intensity of *Mkp1*-positive cells in the cocaine group was significantly higher than in the other two groups in all subregions in the 10-day experiment (*p* < 0.001 for all comparisons) (Fig. 7d). In the 60-day experiment, similar results were observed in all subregions (Coc vs. Con: MO, *p* = 0.003; VO, *p* = 0.01; VLO, *p* = 0.006; LO, *p* = 0.005; DLO, *p* = 0.003; Coc vs. Suc: MO, *p* = 0.009; VO, *p* = 0.015; VLO, *p* = 0.019; LO, *p* = 0.027; DLO, *p* = 0.019) (Fig. 7d). In posterior OFC, after 10 days of self-administration the intensity of *Mkp1*-positive cells was significantly enhanced in the cocaine group in all subregions in comparison to the control group (MO, *p* = 0.007; VLO, *p* < 0.001; AIv, *p* < 0.001; LO, *p* < 0.001; AId, *p* < 0.001; DI, *p* < 0.001). Between cocaine and sucrose groups significant changes were observed in VLO, AIv, LO, AId, and DI (VLO, *p* < 0.001; AIv, *p* < 0.001; LO, *p* = 0.001; AId, *p* < 0.001; DI, *p* = 0.002) (Fig. 7f). After 60 days of self-administration, the intensity of *Mkp1*-positive cells was significantly enhanced in the cocaine group in MO, VLO, AIv, LO, and AId when compared to control and sucrose groups (Coc vs. Con: MO, *p* = 0.002; VLO, *p* = 0.001; AIv, *p* = 0.006; LO, *p* = 0.009; AId, *p* = 0.028; Coc vs. Suc: MO, *p* = 0.001; VLO, *p* < 0.001; AIv, *p* = 0.001; LO, *p* = 0.007; AId, *p* = 0.029) (Fig. 7f).

In summary, compared to the complex changes in the density of *Mkp1*-labeled cells, the alterations in the cellular labeling intensity of *Mkp1-positive* cells were rather straightforward, showing consistent increases in the cocaine groups in most striatal and cortical subregions. Sucrose self-administration had no effect on the cellular intensity, regardless of self-administration duration.

### Topography of neuronal reactivity patterns

Visual inspection of the hybridized tissue sections showed that the cocaine or sucrose self-administration-induced neuronal reactivity patterns did not obey the conventional anatomical boundaries as shown in Fig. 3. We compared the distributional patterns of the reactive neurons to cocaine and sucrose self-administration to establish qualitative and quantitative differences. Using their *X*–*Y* coordinates, all *Mkp1*-positive cells in single sections were plotted and assigned a (color) coding that represented each cell’s labeling intensity, viz. light, medium or intense. This was followed by a warping step to standardized reference sections. Distributional patterns of the cocaine group and sucrose group were compared using SPM analysis.

### Striatum

**Fig. 8:**
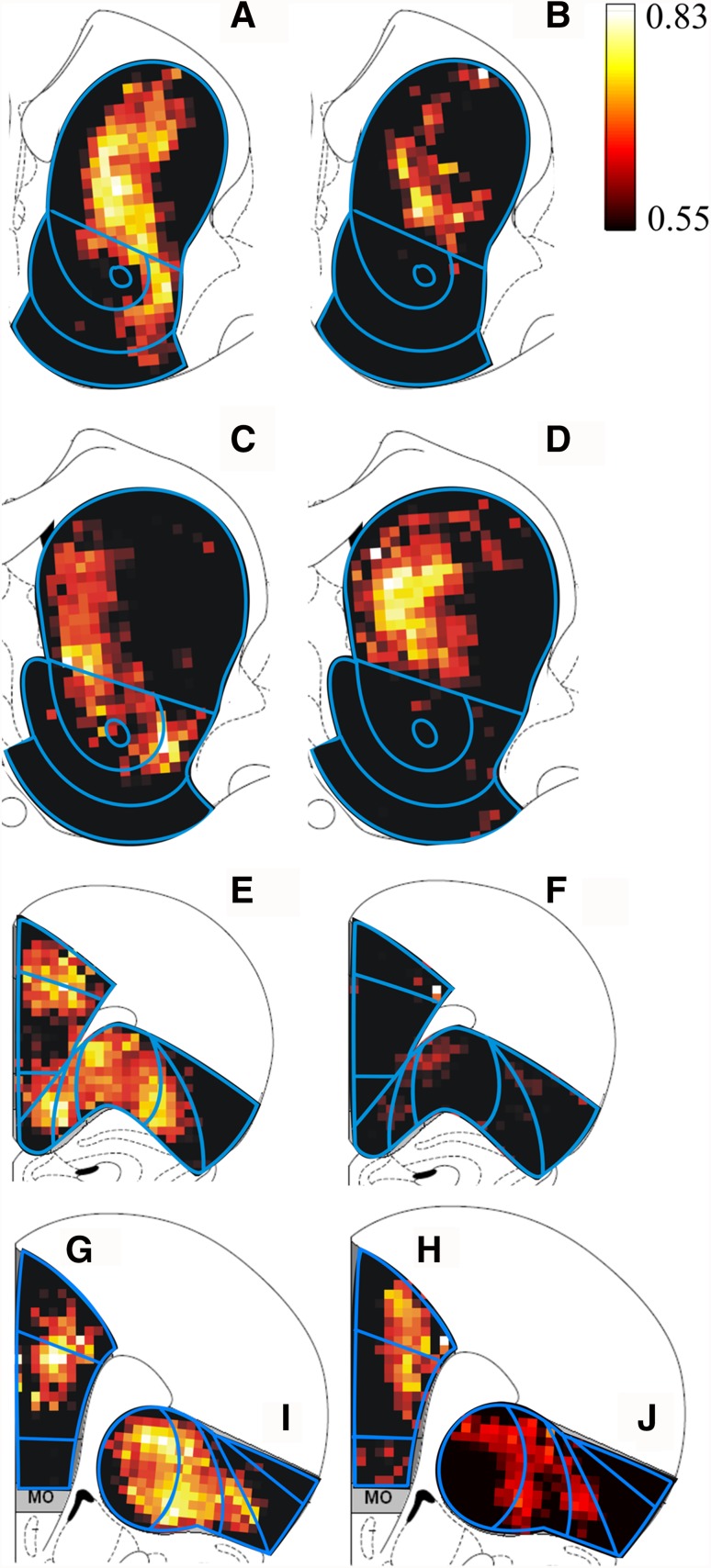
Statistical maps of differences in neuronal activation patterns in frontal cortex and striatum between cocaine- and sucrose-exposed animals. **a, c, e, g, i** Present differences after 10 days self-administration, whereas **b, d, f, h, j** show differences after 60 days self-administration. In striatum, **a** and **b** represent the anterior levels and **c** and **d** the posterior levels. In frontal cortex, **e** and **f** show the anterior levels, and **g** and **h** the posterior levels. Images were thresholded at the following FDR: anterior striatum at 10 days (**a**) FDR = 0.001, at 60 days (**b**) FDR = 0.01. Posterior striatum at 10 days (**c**) FDR = 0.001, at 60 days (**d**) FDR = 0.001. Anterior mPFC + OFC at 10 days (**e**) FDR = 0.001, at 60 days (**f**) FDR = 0.2. Posterior mPFC at 10 days (**g**) FDR = 0.001, at 60 days (**h**) FDR = 0.001. Posterior OFC at 10 days (**i**) FDR = 0.001, at 60 days (**J**) FDR = 0.01. *Color scale* corresponds to the partial correlation coefficient “*r*”. *Superimposed lines* indicate the regions of interest in striatum, mPFC and OFC

Statistical maps of striatum were produced from sections at the two anterior-posterior levels described above. In the anterior striatum, significant differences between the effects of limited (10 days) cocaine and sucrose self-administration were predominantly seen in central parts of DS and laterally in VS (FDR = 0.001, Fig. 8a). Applying the strict limit of FDR = 0.01 showed that, after prolonged (60 days) exposure, the regions displaying significant differences in the anterior striatum had greatly reduced in size (Fig. 8b) compared to the patterns after short-term exposure (Fig. 8a). In contrast, the statistic maps of 10 and 60 days self-administration (cocaine vs. sucrose) in the posterior striatum were dissimilar. After 10 days of administration, significant differences were observed in the medial part of DS, dorsal core and lateral shell (FDR = 0.001, Fig. 8c). However, the significant changes were restricted to a medial sector of DS after 60 days self-administration (FDR = 0.001, Fig. 8d).

### Medial prefrontal and orbitofrontal cortices

In the anterior level, after 10 days of sucrose or cocaine self-administration, major differences were found in the border regions between AC and dorsal PrL, and in a small portion of IL (FDR = 0.001, Fig. 8e). In anterior OFC, the most significant differences between the effects of 10 days of cocaine and sucrose self-administration were seen in three clusters of pixels that were located in medial, central and lateral OFC regions (FDR = 0.001, Fig. 8e). This pattern covered MO, VO, VLO and medial LO, and crossed interregional boundaries; hardly any significant differences were noted in DLO (Fig. 8e). However, after 60 days only minor regions (represented by a few pixels) with significant differences were observed, both in mPFC and OFC, even with a “permissive” FDR setting of 0.2 (Fig. 8f).

Statistical maps in the posterior mPFC showed regions with significant differences primarily in AC and dorsal PrL after both 10 days (Fig. 8g, FDR = 0.001) and 60 days (Fig. 8h, FDR = 0.001) of cocaine self-administration. Compared to 10 days of self-administration, the statistical maps of differences after 60 days of self-administration displayed two marked discrepancies. First, the centers of the pixel clusters (i.e., the yellow-white pixels in Fig. 8g, h) in dorsal mPFC appeared to have shifted from central to more deep layers. Second, more prominent differences between cocaine and sucrose exposure were seen in IL after 60 days exposure (Fig. 8h).

A different kind of contrast may be noted when comparing the statistical maps of anterior with posterior mPFC after 60 days cocaine and sucrose self-administration. Whereas significant changes (at FDR = 0.2) in anterior mPFC were minimal (Fig. 8f), such changes were readily found in posterior mPFC (Fig. 8h) suggesting that extended cocaine exposure induces strong neuronal responses mainly in the posterior levels.

In posterior OFC, most subregions displayed significant differences between the cocaine and sucrose experimental groups after 10 days self-administration, with centers of pixel clusters sitting at the border between VLO and AIv as well as LO and AId (FDR = 0.001, Fig. 8i). After 60 days of self-administration, similar patterns were found, although the areas displaying differences were much smaller (FDR = 0.01, Fig. 8j).

## Discussion

In the present study, we investigated the consequences of cocaine self-administration on neuronal activity in striatum and frontal cortex. The aim of the study was to assess how prolonged drug taking alters patterns of functional activity in these brain structures which may underlie the progression from casual to compulsive drug use. Increased expression of *Mkp1* was found in striatum and frontal cortex after both short- and long-term self-administration of cocaine. These changes were not identical to those reported previously using RT-PCR analysis (Gao et al. 2017). This discrepancy may be caused by the fact that RT-PCR analysis was performed on much larger portions of cortex and striatum than in situ hybridization. Alternatively, it might indicate higher sensitivity of the hybridization technique compared to RT-PCR. In the present study, increased expression of *Mkp1* in striatum was seen in both dorsal and ventral sectors after short-term cocaine exposure; the enhanced reactivity persisted only in dorsal striatum (DS) in rats that had self-administered cocaine for 60 days. In frontal cortex, virtually all medial prefrontal and most orbitofrontal areas showed increased reactivity after 10 days cocaine-administration. However, after 60 days, enhanced expression of *Mkp1* was restricted to caudal parts of medial prefrontal and caudomedial parts of orbitofrontal cortex. In general, the magnitude of changes in *Mkp1* expression was far greater after short-term compared to long-term cocaine self-administration.

Besides a conventional analysis including counting of IEG-expressing neurons and measuring response intensities of individual cells in pre-defined regions of interest, we performed statistical parametric mapping (SPM) of differences between activation patterns after cocaine and sucrose self-administration to establish the distributional features of neuronal responses. The SPM analysis takes into account both the density and intensity of cellular IEG labeling by binning and comparing values for these parameters at the level of pixels. Of course, the size of the pixels will determine the grade of detail in the delineations of local activational differences. In the present experiments, pixel size was set to allow a spatial resolution of approximately the diameter of the anterior commissure, which suffices to distinguish subregions of functionally relevant proportion such as deep and superficial cortical layers or small subregions of nucleus accumbens core and shell. In a similar vein, the outcome of SPM analysis was determined by the face validity-based choice to bin response intensity values into three classes (i.e., light, intermediate and strong immunoreactivity). Using fewer or more classes will affect the grade of detail in the detected differences. Transformation and combination of cellular mappings from individual rats to experimental groups, followed by arithmetic comparison of group differences (e.g., by subtraction) would allow visualization of regional activation differences using, for instance, a LUT scale. However, the prime advantage of the SPM method is that it allows identification of areas with significantly different responses to cocaine and sucrose self-administration. The latter analysis is unbiased, i.e., it does not impose a priori anatomical delineations. Thus, the present data demonstrate changes induced by cocaine and sucrose self-administration at the level of major cortical and striatal regions and subregions thereof.

### Striatum

Limited cocaine self-administration experience (10 days) resulted in increased *Mkp1* cell density and cellular labeling intensity in both ventral striatum (VS) and DS. In the SPM analysis, these changes were represented by a band of pixels indicating significant differences between cocaine and sucrose self-administration that ran from dorsomedial to ventrolateral striatum. In contrast, after prolonged (60 days) exposure to cocaine and sucrose this band was limited to DS. Whereas increased *Mkp1* cell density was observed in both DS and VS after short-term cocaine self-administration, this response persisted only in DS after long-term cocaine use. In addition, in VS the augmented intensity of cell labeling in nucleus accumbens shell was found to be lower after 60 days compared to 10 days.

Activation of VS and DS after short-term cocaine self-administration as opposed to more selective activation of DS after long-term cocaine use, hints at the notion that striatal control over drug intake mainly involves DS with prolonged drug use (Everitt and Robbins 2005, 2016). The posterior dorsomedial part of striatum has been implicated in action–outcome learning, typified by action selection that is sensitive to changes in reward value, and hence, is flexible and goal-directed (Yin and Knowlton 2006; Yin et al. 2008; Shiflett and Balleine 2011). Indeed, a clear association has been reported between the action–outcome learning phase in a behavioral task and cellular activity in the dorsomedial striatum (Stalnaker et al. 2010; Thorn et al. 2010; Kim et al. 2013). Together with the present findings, this suggests that corticostriatal networks involving the dorsomedial, associative compartment of striatum play a role in the initial phases of the development of drug addiction. In later phases, striatal control over cocaine seeking is thought to be governed by the dorsolateral striatum (Vanderschuren et al. 2005; Belin and Everitt 2008; Pierce and Vanderschuren 2010; Zapata et al. 2010; Jonkman et al. 2012a; Murray et al. 2012). Interestingly, the present data demonstrate that in DS increased cocaine-specific activity is restricted to medial and central locations after prolonged experience with cocaine. The dorsocentral striatum is likely important in regulating cocaine intake (Hollander et al. 2010). It has been proposed to mediate sequencing of actions that is necessary for constructing complex action patterns—so called chunking (Barnes et al. 2010; Graybiel and Grafton 2015)—a prerequisite for goal-directed behavior to shift to habitual responding (Shiflett and Balleine 2010). The present patterns of neuronal activity are likely related to this process, whereby the act of responding for cocaine becomes a chunked, ingrained activity that ultimately results in automated, habitual cocaine taking. Somewhat contrary to our hypothesis, we did not find changes in *Mkp1* expression in dorsolateral striatal parts implicated in habitual drug seeking. In this study, we did not explicitly test whether cocaine self-administration had become habitual or resistant to punishment to avoid any influence of punishment on IEG expression. For that reason, we can only speculate on whether the 60 days of responding for cocaine under an FR1 schedule of reinforcement indeed resulted in habitual behavior that depends on the dorsolateral striatum. On one hand, dopamine neurotransmission in the dorsolateral striatum has been shown to be involved in responding for cocaine under fixed-ratio schedules of reinforcement (Veeneman et al. 2012, 2015; Willuhn et al. 2012). On the other hand, it has also been suggested that the dorsolateral striatum only becomes involved in cocaine self-administration when high rates of behavior are required to procure the drug (Murray et al. 2012). In addition, the development of punishment-resistant cocaine seeking has been shown to require exposure to self-administration episodes in which animals can self-administer large quantities of the drug during prolonged experimental sessions (Vanderschuren and Everitt 2004; Jonkman et al. 2012b). Even under these conditions, compulsive patterns of drug seeking and taking may only occur in a subset of animals (Deroche-Gamonet et al. 2004; Pelloux et al. 2007, 2012). Thus, we cannot be sure that the behavior of our cocaine self-administering animals after 60 days of drug taking had gained a habitual, or perhaps compulsive quality that relies on the dorsolateral striatum. Rather, the involvement of dorsocentral regions after 60 days of cocaine self-administration suggests that the self-administration behavior had become ‘chunked’, and was therefore in the process of becoming automated.

### Medial prefrontal cortex

Loss of cognitive control over drug use is thought to occur as a consequence of drug-induced dysfunction of prefrontal and orbitofrontal cortex (Jentsch and Taylor 1999; Goldstein and Volkow 2011; Everitt and Robbins 2016). In the present experiments, we hypothesized such a process to result in neuronal reactivity patterns that differed between short- and long-term self-controlled cocaine intake. Indeed, all three prefrontal regions (i.e., AC, PrL and IL) showed increased activity after 10 days of cocaine self-administration, whereas much smaller rises in activity were seen after 60 days, restricted to posterior parts of PrL and IL. The neuronal activity in PrL is likely related to the role that this cortical region plays in goal-directed behavior, i.e., outcome value-controlled responding (Chudasama et al. 2003; Killcross and Coutureau 2003; Balleine and O’Doherty 2010). The present data indicate that this model-based control over instrumental responding (Daw et al. 2011) is strong after limited drug exposure, whereas PrL control over drug seeking weakens after long-term repeated exposure to the drug. The IL has been implicated in the development of habitual responding by organizing action sequences into habits (Killcross and Coutureau 2003; Smith and Graybiel 2013) and such activity may be reflected by the shift in neuronal reactivity from anterior to posterior levels in IL after long-term cocaine self-administration. Activity in AC has been demonstrated to be important for the temporal organization of action chains in which executive attention processes play a crucial role (Passetti et al. 2002; Chudasama et al. 2003; Heidbreder and Groenewegen 2003). Experimental findings on conditioning processes involving cocaine are in agreement with the purported function in attention for AC in humans as well as rats (Weissenborn et al. 1997; Garavan et al. 2000; Neisewander et al. 2000; McLaughlin and See 2003; Baeg et al. 2009; Goldstein et al. 2009). Our SPM analysis showed neuronal reactivity to engage a larger part of AC in posterior mPFC after long-term cocaine self-administration. This may reflect enhanced attention and learning or perhaps a form of attentional bias (Luijten et al. 2011; Marhe et al. 2013; Kilts et al. 2014).

### Orbitofrontal cortex

Augmented neuronal reactivity after short-term cocaine self-administration was observed in both medial and lateral OFC, except the most lateral portion of the OFC (i.e., the DLO) in the present study. After long-term cocaine exposure increased neuronal reactivity was largely restricted to posterior levels of VLO and LO (and AIv), whereas anterior lateral and medial parts of OFC no longer were responsive. These differential effects suggest diverse changes in response regulation related to dissociable functions of OFC subregions (Elliott et al. 2000; Windmann et al. 2006; Burton et al. 2015 and references therein; see also Rudebeck and Murray 2011a, b). Although both medial and lateral OFC have been implicated in responding to cocaine-associated cues (Fuchs et al. 2004; Stalnaker et al. 2006; Goldstein et al. 2007; Kantak et al. 2013), we are only beginning to understand the precise functional involvement of the individual OFC subregions in behavioral control (Noonan et al. 2010; Mar et al. 2011; Burton et al. 2014).

The OFC is thought to encode the value of anticipated rewards or outcome of actions. Prior cocaine use has been shown to affect the sensitivity of OFC neurons to value of predicted outcome required for behavioral response flexibility (Lucantonio et al. 2014). The current findings of increased neuronal activity in OFC after 10 days of cocaine self-administration may represent the involvement of value sensitivity in drug taking. According to our SPM analysis, increased activity engaged the entire OFC except the DLO region. This response dwindles as experience with cocaine self-administration grows and after 60 days, enhanced activity was reduced to a minimum in anterior levels, whereas posteriorly an activity pattern was seen that essentially resembled the distribution at 10 days albeit with lower significance (i.e., FDR rates). Interestingly, after 60 days, sucrose exposure resulted in significantly increased neuronal activity in LO and no apparent effect of cocaine use, inducing a significant difference between the cocaine- and sucrose-exposed experimental groups. These findings point to a strong influence on behavioral control of anticipated outcome value after 10 days of cocaine self-administration and a greatly reduced impact of value encoding in response regulation after 60 days exposure. High neuronal activity levels in OFC have been associated with goal-directed actions, whereas lower activity was found with habitual responding (Gremel and Costa 2013). The reduced rise in activity in OFC as seen in the present experiments, suggests that OFC involvement is waning and that responding is becoming routinized.

### Sucrose and cocaine self-administration

In contrast to cocaine, sucrose self-administration did not induce major changes in neuronal reactivity. Only after long-term access to sucrose increases were found in *Mkp1* cell density, which were restricted to anterior dorsal mPFC and lateral OFC. Interestingly, in the same regions the effects of cocaine exposure had disappeared after long-term self-administration. These findings suggest that different cortical areas are involved in instrumental conditioning for a drug or natural reward. Alternatively, temporal differences in engaging the cortical regions in the conditioning process may play a role and the present observation of sucrose-associated effects after long-term exposure might represent an initial stage in sucrose-induced neuroadaptive changes. Indeed, in mPFC as well as striatum, neuroadaptive changes in protein expression have been reported after limited and excessive intake of sucrose (Van den Oever et al. 2006; Ahmed et al. 2014). A late role for mPFC and OFC would be in line with lesion experiments showing no effect on acquisition of sucrose self-administration, whereas cocaine self-administration was impaired (Weissenborn et al. 1997). In a reinstatement paradigm, Liu et al. (2013) showed distinct neuronal encoding of cocaine- or sucrose-associated stimuli in striatum and mPFC subregions, although the same areas were activated by the differently conditioned cues. In addition, Levy et al. (2007) found no effect of mPFC stimulation on sucrose-rewarded behavior in contrast to cocaine-rewarded behavior.

## Conclusions

The present findings provide a neuronal activation correlate of the proposed change in functional involvement of prefrontal cortex, and dorsal and ventral striatum in prolonged drug intake. After long-term cocaine self-administration, neuronal activity was largely confined to the dorsomedial and dorsocentral striatum. In the frontal lobe, much less activation was seen after long-term compared to short-term cocaine exposure. The observed changes in neuronal activity from short-term to long-term drug exposure might reflect drug taking becoming routinized and in the process of becoming habitual.

## Electronic supplementary material

Below is the link to the electronic supplementary material.

**Table S1** Overview of cellular density (Density, i.e., number of cells per mm^2^) and cellular labeling intensity (intensity, i.e., OD) of *Mkp1*-positive cells in individual subregions of striatum, mPFC, and OFC. (PDF 84 KB)

## Abbreviations

*Mkp1*: Mitogen-activated protein kinase phosphatase 1
IEG: Immediate early gene
ISH: In situ hybridization
SPM: Statistical parametric mapping
DS: Dorsal striatum
VS: Ventral striatum
Tu: Olfactory tubercle
mPFC: Medial prefrontal cortex
OFC: Orbitofrontal cortex
AC: Anterior cingulate cortex
PrL: Prelimbic cortex
IL: Infralimbic cortex
MO: Medial orbitofrontal cortex
VO: Ventral orbitofrontal cortex
VLO: Ventral lateral orbitofrontal cortex
LO: Lateral orbitofrontal cortex
DLO: Dorsolateral orbitofrontal cortex
AIv: Ventral agranular insular cortex
AId: Dorsal agranular insular cortex
DI: Dysgranular insular cortex
