# Supplementary material for: A neuronal activation correlate in striatum and prefrontal cortex of prolonged cocaine intake
Source: Brain Struct Funct. 2017 Apr 9;222(8):3453–75. doi: 10.1007/s00429-017-1412-4 (PMC5676843; doi:10.1007/s00429-017-1412-4)
Supplement: Supplementary file 1 — Table S1 Overview of cellular density (Density, i.e., number of cells per mm2) and cellular labeling intensity (intensity, i.e., OD) of Mkp1-positive cells in individual subregions of striatum, mPFC, and OFC. (PDF 84 KB) [file 429_2017_1412_MOESM1_ESM.pdf]

Title: A neuronal activation correlate in striatum and prefrontal cortex of prolonged cocaine intake

Journal Name: Brain Structure and Function

Authors: Ping Gao[1], Jan C De Munck[2], Jules H.W. Limpens[3], Louk J.M.J. Vanderschuren[4], Pieter Voorn[1]

[1] Department of Anatomy and Neurosciences, Neuroscience Campus Amsterdam, VU University Medical Center, Amsterdam, The Netherlands

[2] Department of Physics and Medical Technology, VU University Medical Center, De Boelelaan 1118, 1081 HZ Amsterdam, The Netherlands.

[3] Brain Center Rudolf Magnus, Department of Translational Neuroscience, University Medical Center Utrecht, Utrecht, The Netherlands

[4] Department of Animals in Science and Society, Division of Behavioural Neuroscience, Faculty of Veterinary Medicine, Utrecht University, Utrecht, The Netherlands

Author for Correspondence:

Pieter Voorn, Ph.D

Email: p.voorn@vumc.nl

Table S1

Region	Sub-region	Group	Short term (10 days)		Long term (60 days)	
			**Density**	**Intensity**	**Density**	**Intensity**
Striatum (Anterior)	DS	Control	42 ± 7.29	0.028 ± 0.0012	50 ± 4.17	0.023 ± 0.0024
		Sucrose	35 ± 3.84	0.031 ± 0.0013	52 ± 6.09	0.028 ± 0.0015
		Cocaine	102 ± 5.61	0.039 ± 0.0012	103 ± 9.09	0.034 ± 0.0014
	Core	Control	20 ± 3.21	0.032 ± 0.0023	74 ± 6.21	0.025 ± 0.0026
		Sucrose	17 ± 2.29	0.031 ± 0.0012	75 ± 8.44	0.029 ± 0.0020
		Cocaine	62 ± 5.58	0.044 ± 0.0031	97 ± 6.68	0.037 ± 0.0013
	Shell	Control	26 ± 4.74	0.031 ± 0.0017	82 ± 8.53	0.026 ± 0.0029
		Sucrose	23 ± 2.66	0.033 ± 0.0016	82 ± 12.64	0.029 ± 0.0012
		Cocaine	55 ± 4.20	0.041 ± 0.0023	106 ± 11.90	0.036 ± 0.0022
	Tu	Control	26 ± 3.36	0.030 ± 0.0017	167 ± 15.28	0.025 ± 0.0029
		Sucrose	31 ± 5.85	0.032 ± 0.0018	171 ± 21.30	0.028 ± 0.0012
		Cocaine	66 ± 6.89	0.038 ± 0.0014	173 ± 30.66	0.029 ± 0.0008
Striatum (Posterior)	DS	Control	38 ± 5.27	0.034 ± 0.0014	63 ± 3.28	0.028 ± 0.0011
		Sucrose	50 ± 2.77	0.037 ± 0.0008	71 ± 8.73	0.029 ± 0.0010
		Cocaine	118 ± 10.58	0.049 ± 0.0028	93 ± 7.33	0.043 ± 0.0024
	Core	Control	20 ± 2.33	0.039 ± 0.0026	50 ± 8.05	0.030 ± 0.0015
		Sucrose	27 ± 2.84	0.041 ± 0.0023	60 ± 10.08	0.029 ± 0.0017
		Cocaine	53 ± 3.15	0.058 ± 0.0037	64 ± 5.08	0.044 ± 0.0022
	Shell	Control	24 ± 3.30	0.036 ± 0.0020	49 ± 6.03	0.032 ± 0.0014
		Sucrose	26 ± 2.06	0.040 ± 0.0011	54 ± 9.37	0.030 ± 0.0017
		Cocaine	51 ± 3.64	0.055 ± 0.0030	66 ± 5.93	0.041 ± 0.0019
	Tu	Control	26 ± 6.45	0.037 ± 0.0037	102 ± 17.01	0.027 ± 0.0011
		Sucrose	31 ± 2.87	0.037 ± 0.0020	123 ± 18.42	0.025 ± 0.0008
		Cocaine	50 ± 5.03	0.044 ± 0.0028	145 ± 15.26	0.035 ± 0.0018
mPFC (Anterior)	AC	Control	157 ± 12.59	0.033 ± 0.0018	394 ± 17.39	0.036 ± 0.0012
		Sucrose	176 ± 14.88	0.035 ± 0.0022	481 ± 40.56	0.039 ± 0.0015
		Cocaine	260 ± 14.19	0.052 ± 0.0014	364 ± 20.35	0.045 ± 0.0020
	PrL	Control	124 ± 9.28	0.037 ± 0.0024	329 ± 14.41	0.037 ± 0.0012
		Sucrose	146 ± 10.49	0.039 ± 0.0025	388 ± 33.42	0.039 ± 0.0015
		Cocaine	197 ± 10.00	0.059 ± 0.0012	326 ± 19.84	0.045 ± 0.0016

Table S1 continued

Region	Sub-region	Group	Short term (10 days)		Long term (60 days)	
			**Density**	**Intensity**	**Density**	**Intensity**
	IL	Control	122 ± 12.36	0.040 ± 0.0015	266 ± 12.61	0.038 ± 0.0015
		Sucrose	136 ± 11.20	0.040 ± 0.0030	302 ± 24.47	0.040 ± 0.0016
		Cocaine	207 ± 12.47	0.057 ± 0.0022	290 ± 19.18	0.048 ± 0.0017
mPFC (Posterior)	AC	Control	148 ± 11.16	0.037 ± 0.0019	302 ± 8.32	0.037 ± 0.0027
		Sucrose	176 ± 10.97	0.040 ± 0.0011	304 ± 22.30	0.034 ± 0.0021
		Cocaine	232 ± 19.19	0.052 ± 0.0011	344 ± 12.18	0.043 ± 0.0011
	PrL	Control	106 ± 13.94	0.041 ± 0.0025	260 ± 12.67	0.038 ± 0.0021
		Sucrose	125 ± 8.13	0.043 ± 0.0011	247 ± 16.09	0.035 ± 0.0013
		Cocaine	183 ± 7.35	0.056 ± 0.0008	309 ± 8.68	0.043 ± 0.0015
	IL	Control	69 ± 13.28	0.044 ± 0.0030	139 ± 12.79	0.035 ± 0.0020
		Sucrose	66 ± 5.61	0.047 ± 0.0017	159 ± 17.36	0.032 ± 0.0012
		Cocaine	101 ± 4.30	0.056 ± 0.0010	213 ± 11.60	0.041 ± 0.0014
OFC (Anterior)	MO	Control	156 ± 14.00	0.038 ± 0.0022	337 ± 11.52	0.038 ± 0.0013
		Sucrose	161 ± 11.63	0.039 ± 0.0028	355 ± 28.06	0.039 ± 0.0011
		Cocaine	272 ± 15.23	0.060 ± 0.0015	347 ± 22.39	0.047 ± 0.0022
	VO	Control	178 ± 13.33	0.036 ± 0.0022	467 ± 17.44	0.039 ± 0.0015
		Sucrose	177 ± 9.26	0.040 ± 0.0028	554 ± 53.39	0.039 ± 0.0012
		Cocaine	299 ± 18.73	0.056 ± 0.0013	429 ± 22.95	0.046 ± 0.0019
	VLO	Control	229 ± 14.63	0.035 ± 0.0017	455 ± 14.53	0.042 ± 0.0020
		Sucrose	238 ± 17.44	0.037 ± 0.0025	564 ± 44.29	0.044 ± 0.0016
		Cocaine	328 ± 20.83	0.056 ± 0.0016	474 ± 20.13	0.053 ± 0.0026
	LO	Control	208 ± 18.50	0.036 ± 0.0019	378 ± 10.04	0.044 ± 0.0020
		Sucrose	222 ± 13.31	0.041 ± 0.0028	483 ± 30.70	0.046 ± 0.0018
		Cocaine	298 ± 19.47	0.059 ± 0.0020	405 ± 11.98	0.056 ± 0.0029
	DLO	Control	118 ± 14.12	0.033 ± 0.0017	351 ± 13.02	0.037 ± 0.0011
		Sucrose	131 ± 9.95	0.037 ± 0.0026	415 ± 26.97	0.038 ± 0.0013
		Cocaine	159 ± 12.55	0.053 ± 0.0020	323 ± 17.03	0.045 ± 0.0020

Table S1 continued

Region	Sub-region	Group	Short term (10 days)		Long term (60 days)	
			Density	Intensity	Density	Intensity
OFC (Posterior)	MO	Control	55 ± 11.89	0.037 ± 0.0029	181 ± 15.57	0.031 ± 0.0017
		Sucrose	59 ± 3.98	0.041 ± 0.0024	213 ± 22.36	0.030 ± 0.0019
		Cocaine	105 ± 5.27	0.049 ± 0.0007	247 ± 12.94	0.041 ± 0.0011
	VLO	Control	160 ± 20.49	0.037 ± 0.0021	351 ± 13.12	0.039 ± 0.0019
		Sucrose	150 ± 13.99	0.040 ± 0.0012	366 ± 21.85	0.038 ± 0.0027
		Cocaine	250 ± 7.24	0.055 ± 0.0017	426 ± 13.37	0.052 ± 0.0015
	AIv	Control	163 ± 23.99	0.039 ± 0.0024	357 ± 10.36	0.041 ± 0.0021
		Sucrose	171 ± 8.99	0.042 ± 0.0017	366 ± 15.68	0.039 ± 0.0026
		Cocaine	253 ± 7.28	0.061 ± 0.0017	407 ± 11.32	0.051 ± 0.0010
	LO	Control	125 ± 19.71	0.041 ± 0.0030	299 ± 12.21	0.041 ± 0.0021
		Sucrose	141 ± 8.26	0.042 ± 0.0013	314 ± 11.08	0.040 ± 0.0029
		Cocaine	195 ± 7.29	0.059 ± 0.0022	341 ± 10.58	0.051 ± 0.0007
	AId	Control	96 ± 16.53	0.037 ± 0.0020	257 ± 13.09	0.037 ± 0.0022
		Sucrose	111 ± 5.81	0.039 ± 0.0007	281 ± 15.49	0.037 ± 0.0021
		Cocaine	111 ± 4.00	0.054 ± 0.0011	292 ± 13.19	0.045 ± 0.0012
	DI	Control	107 ± 16.14	0.035 ± 0.0021	309 ± 11.93	0.038 ± 0.0019
		Sucrose	129 ± 11.86	0.038 ± 0.0011	289 ± 18.62	0.034 ± 0.0026
		Cocaine	115 ± 7.84	0.047 ± 0.0011	272 ± 17.65	0.040 ± 0.0013
